# *Hyalangiumruber* sp. nov, characterization of a novel myxobacterium strain s54d21 and their secondary metabolites

**DOI:** 10.3389/fmicb.2024.1369499

**Published:** 2024-03-08

**Authors:** Yi Zang, Xianjiao Zhang, Zhe Wang, Qingyi Tong, Yang Zhou, Qing Yao, Honghui Zhu

**Affiliations:** ^1^Key Laboratory of Agricultural Microbiomics and Precision Application (MARA), Guangdong Provincial Key Laboratory of Microbial Culture Collection and Application, Key Laboratory of Agricultural Microbiome (MARA), State Key Laboratory of Applied Microbiology Southern China, Institute of Microbiology, Guangdong Academy of Sciences, Guangzhou, China; ^2^College of Horticulture, South China Agriculture University, Guangzhou, China; ^3^School of Pharmacy, Tongji Medical College, Huazhong University of Science and Technology, Wuhan, China

**Keywords:** myxobacteria, *Hyalangium ruber*, secondary metabolite, structure elucidation, cytotoxicity

## Abstract

Myxobacteria are special bacteria with wide adaptability, which are rich sources of structurally diverse natural products with intriguing biological properties. Here, a gram-negative myxobacterium strain s54d21^T^ was isolated from the sediment of a wetland park in China using the *Escherichia coli* baiting method. Based on 16S rRNA gene sequence and genomic data, the strain was demonstrated to be a novel species of a rare genus *Hyalangium*, designated *Hyalangium ruber* sp. nov (type strain s54d21^T^ = GDMCC 1.1945^T^ = JCM 39263^T^). The subsequent chemical investigation of the strain s54d21^T^ led to the isolation of three rare 3,5,6-trisubstituted 2(1*H*)-pyrazinones, namely, hyalanones A–C (**1**–**3**), together with a known macrolactin A (**4**). Those new structures and their absolute configurations were unambiguously assigned by extensive analyses of spectroscopic data and density functional theory (DFT) calculations. In biological assays, compound **4** exhibited moderate cytotoxic activities against human cell lines RKO, A549, and NCM460 with IC_50_ values ranging from 27.21 to 32.14 *μ*M.

## 1 Introduction

Myxobacteria are a class of gram-negative eubacteria widely found in terrestrial and aquatic ecosystem. They are known for their complex lifecycle and social behavior that are probably mediated through secondary metabolites (Kaiser, 2003; Shrivastava and Sharma, 2021; Saggu et al., 2023). In past four decades, numbers of natural products from myxobacteria have been characterized with novel and complex structures, including polyketides, non-ribosomal peptides, and hybrids (Wenzel and Müller, 2009; Herrmann et al., 2017; Bader et al., 2020; Saggu et al., 2023). These secondary metabolites exhibit diverse biological properties, such as cytotoxic, antifungal, antibacterial, antiviral, antimalarial, and immunosuppressive effects (Wenzel and Müller, 2009; Schäberle et al., 2014; Dehhaghi et al., 2018; Bhat et al., 2021). Hence, myxobacteria are considered as important and enormous sources for structurally diverse and bioactive natural products that can be explored for drug discovery.

Although amount of myxobacteria have been identified in past two decades, there are numerous undiscovered or uncultivated myxobacteria. The genus *Hyalangium* belonging to Myxococcaceae family was initially established by Reichenbach (2005), while the only member *H. minutum* was isolated from a mountain soil with decayed plants before 2023. Continuous chemical research studies of *H. minutum* found a series of bioactive compounds, including hyaladione with significant cytotoxic and antimicrobial activities and unusual siderophores, namely, hyalachelins A–C (Okanya et al., 2012; Nadmid et al., 2014; Okanya et al., 2014; Surup et al., 2018). Until recently, two novel species of *H. versicolor* and *H. gracilis* were characterized and reclassified by Zhang et al. (2023).

In this study, the strain s54d21^T^ was isolated from a sample of wetland sediment as a novel member of *Hyalangium* classified by using the polyphasic approach. To search for novel and bioactive natural products from this strain, the chemical investigation of the culture broth of *Hyalangium ruber* sp. nov was performed, leading to the identification of three 2(1*H*)-pyrazinone derivatives, namely, hyalanones A–C (**1**–**3**), along with a known compound macrolactin A (**4**). The planar structures and absolute stereochemistry of those new isolates were unambiguously established by extensive spectroscopic methods and theoretical electronic circular dichroism (ECD) calculations with time-dependent DFT (TDDFT) methods. Hyalanones A–C (**1**–**3**) were assigned as rare 3,5,6-trisubstituted 2(1*H*)-pyrazinone analogs that were probably biosynthesized from valine and alanine or threonine, respectively. The known compound **4** was determined by comparing of NMR data and HRESIMS spectrum with literatures (Gustafson et al., 1989; He et al., 2013). Herein, we report the identification of the myxobacterium strain, isolation and structure elucidation of new compounds, and their biological assay.

## 2 Materials and methods

### 2.1 Isolation and cultivation of the myxobacteria strain

Strain s54d21^T^ was isolated from a sediment sample collected from Xinghu National Wetland Park (N 23°4′54″, E 112°28′17″) in Guangdong Province of China. The strain was isolated following the baiting approach with *E. coli* as prey on water agar (0.1% CaCl_2_∙2H_2_O, 20 mM HEPES, 1.5% agar), where small portions of soil samples were placed adjacent to the *E. coli* spot. Until swarming colonies or fruiting bodies observed after incubation at 30°C, purification of the isolate was subsequently completed by repeatedly transferring onto fresh VY/2 agar (0.5% dried baker’s yeast, 0.1% CaCl_2_∙2H_2_O, 1.5% agar). Finally, the pure strain characterized orange-pigment fruiting bodies on agar and was deposited in Guangdong Microbial Culture Collection Center (GDMCC, No. 1.1945) and Japan Collection of Microorganisms (JCM, no. 39263).

### 2.2 Morphological and physiological analyses

The swarm morphology was observed under a stereomicroscope (Olympus SZX10, Japan). The vegetative cells and myxospores were investigated by using scanning electron microscope (SEM, Hitachi S-3000 N, Japan) and the phase contrast microscope (ZEISS Axioscope 5, Germany), respectively. The Gram reaction was determined using a Gram staining kit (Huankai, China), while Conga red staining was performed as described by McCurdy (1969). The oxidase test was determined using a commercial strip (Huankai, China), while the catalase test was performed using 3% (v/v) H_2_O_2_. The growth temperature range was determined on VY/2 agar at different temperatures (4, 10, 15, 20, 25, 30, 37, 40, and 42°C). The pH range (4.0–10.0, at intervals of 1 pH unit) for growth was tested on VY/2 agar at 30°C for 14 days and buffered with 100 mM citrate/sodium citrate (pH 4.0–5.0), HEPES (pH 6.0–8.0), and Tris (pH 9.0–10.0). Salt tolerance was evaluated by growing the isolated on VY/2 agar plates supplemented with NaCl to concentrations of 0.5, 1, 1.5, 2, and 3% (m/v). Susceptibility to antibiotics was investigated on VY/2 agar supplemented with various antibiotics at the final concentration of 50 μg/mL at 30°C for 14 days. Sixteen antibiotics were selected for strain s54d21^T^: ampicillin, apramycin, spectinomycin, polymyxin B, neomycin, bacitracin B, gentamicin, tetracycline, erythromycin, oxytetracycline, chloramphenicol, nalidixic acid, trimethoprim, streptomycin, rifampin, and kanamycin. Anaerobic growth was tested on VY/2 agar plates in an anaerobic jar at 30°C for 14 days. Hydrolysis of starch, skimmed milk, colloidal chitin, and carboxymethycelluose (CMC) was assessed on tryptone agar (0.2% tryptone, 0.05% MgSO_4_, 0.01% CaCl_2_∙2H_2_O, 1.5% agar; pH 7.2) at the final concentration of 1.0% (m/v), respectively. Hydrolysis of Tween 20 and 80 was tested on tryptone agar supplemented with 1.0% (v/v) Tween 20 and 80, respectively. Additional enzyme activities were tested using the API ZYM and 20NE kits (bioMérieux, France), according to the manufacturer’s instructions.

### 2.3 16S RNA analysis identification and phylogenetic analysis

Genomic DNA of strain s54d21^T^ was extracted by using Genomic DNA Isolation Kit (Magen, China). The 16S rRNA gene sequence was amplified using the universal primers 27F/1492R (Weisburg et al., 1991). The PCR products were sequenced by Shanghai Majorbio Biopharm Technology Co., Ltd. Sequence alignment was performed at online EZBioCloud server (Yoon et al., 2017a) and NCBI. Phylogenetic tree based on the 16S rRNA gene sequences was reconstructed using software MEGA X (Kumar et al., 2018) with the maximum likelihood (ML) method (Felsenstein, 1981) under the Kimura’s two-parameter model (Kimura, 1980). Bootstrap analysis was conducted based on 1,000 replicates (Hoang et al., 2018).

### 2.4 Genome sequencing and bioinformatics analysis

Genomic sequencing was carried out using an Illumina Hiseq platform at Shanghai Majorbio Biopharm Technology Co., Ltd. The obtained reads were assembled into contigs using SPAdes v3.11.1 (Bankevich et al., 2012). Genome contamination and completeness were assessed using CheckM tool (Parks et al., 2015). Genome was annotated using the Prokka (Seemann, 2014) and RAST server (Aziz et al., 2008). The biosynthetic gene clusters of secondary metabolites were predicted using antiSMASH 6.0 with the default settings (Blin et al., 2021). The carbohydrate activities were identified using the dbCAN2 meta server (Zhang et al., 2018). Average nucleotide identity (ANI) and digital DNA–DNA hybridization (dDDH) values among strain s54d21^T^ and the closely related type strains were calculated using an online ANI calculator and the Genome-to-Genome Distance Calculator 3.0 (Meier-Kolthoff et al., 2013; Yoon et al., 2017b). Phylogenomic tree was constructed using the up-to-data bacterial core gene (UBCG) pipeline based on 92 core housekeeping genes (Na et al., 2018).

### 2.5 Chemotaxonomic characterization

Respiratory quinones of the strain s54d21^T^ were extracted and analyzed as previously described using HPLC (Agilent 1260) (Collins et al., 1977). For the fatty acid detection, strain s54d21^T^ and its closely related type strains were incubated on VY/2 agar plates at 30°C for a week. Fatty acids were extracted and identified according to the described protocol using a gas chromatography mass spectrometry (GCMS, Agilent 7890B-5977B) (Gemperlein et al., 2014).

### 2.6 Chemical investigation of the myxobacteria strain

#### 2.6.1 General experimental procedures

The UV, IR, and ECD spectra were obtained on a Jobin Yvon LabRAM HR800 instrument, a Bruker Vertex 70 instrument, and a JASCO-810 ECD spectrometer, respectively. Optical rotation values of new compounds were tested with a Rudolph Autopol IV automatic polarimeter. NMR spectra were recorded on a Bruker Ascend 600 MHz spectrometer, with ^1^H and ^13^C NMR chemical shifts referenced to the solvent or solvent impurity peaks for CD_3_OD (*δ*_H_ 3.31 and *δ*_C_ 49.0). HRESIMS data were acquired using electrospray ionization (ESI) with a Thermo Fisher Orbitraq Exploris 120 mass spectrometer. Semi-preparative HPLC utilized a SHIMADZU Prominence LC-20AT quaternary system with a UV–VIS detector using a YMC-pack ODS-A column (5 μm, 10 × 250 mm). Column chromatography (CC) was performed using ODS (50 μm, YMC, Japan). Thin-layer chromatography (TLC) was performed with silica gel 60 GF254 (Yantai Chemical Industry Research Institute, China).

#### 2.6.2 Fermentation, extraction, and isolation

##### 2.6.2.1 Extraction and isolation

The strain was maintained on VY/2 agar at 30°C for 5 days, which was used as the seed culture. The strain was cultured in 10 L MD1G liquid medium (0.3% tryptone, 5.0% starch, 0.02% MgSO_4_∙2H_2_O, 0.07% CaCl_2_∙2H_2_O) on a rotary shaker (180 rpm) at 30°C for 10 days. Amberliter XAD-16 (2%, v/v) was added to the liquid medium on the fourth day (Okanya et al., 2012, 2014). After incubation, XAD and cell mass were harvested by centrifugation and filtration, which was washed using 10 L acetone and 10 L methanol sequentially at room temperature to yield 600 mg of crude extract.

The crude extract was fractioned by column chromatography (CC) over ODS with a gradient elution using MeOH/H_2_O gradient (25 to 100%, v/v) to afford 9 fractions (Fr.1–Fr.9). Compound **1** (6.4 mg) was isolated from Fr.3–3 via semi-preparative HPLC (25% MeCN/H_2_O for 45 min; flow rate: 3.0 mL/min; **1**: *t*_R_ = 38.0 min). Compounds **2** (0.9 mg) and **3** (1.0 mg) were further purified from Fr.3–1-3 by HPLC (20% MeCN/H_2_O for 30 min; flow rate: 3.0 mL/min; **2**: *t*_R_ = 9.9 min; **3**: *t*_R_ = 24.6 min). Fr.4–4 was further purified by HPLC (40% MeCN/H_2_O for 35 min; flow rate: 3.0 mL/min; **4**: *t*_R_ = 29.8 min) to get compound **4** (3.5 mg).

#### 2.6.3 Spectroscopic data of new compounds

Hyalanone A (**1**): amorphous solid; UV (MeOH) *λ*_max_ nm (log *ε*): 231 (4.2), 331 (4.0); IR (NaCl) *ν*_max_ cm^−1^: 3364, 2,966, 2,923, 2,869, 2,852, 1,648, 1,533, 1,466, 1,389, 1,347, 1,294, 1,212, 1,083, 1,036, 960, 816, 650, 623, 546, and 522; ^1^H and ^13^C NMR data (CD_3_OD) (see Table 1); HRESIMS m/z 203.1155 [M + Na]^+^ (calcd for C_10_H_16_N_2_ONa, 203.1160).

**Table 1:** Differential phenotypic and physiological characteristics of the strain s54d21^T^ and the closely related type strains.

Characteristics	1	2	3	4
Temperature growth range (°C)	20–40	15–40	20–42	20–37
pH tolerance	4.0–10.0	5.0–10.0	4.0–10.0	4.0–10.0
*β-*Galactosidase	−	+	−	−
Cystine arylamidase	−	+	+	+
Trypsin	−	−	+	+
*α*-Chymotrypsin	−	−	+	+
*β*-Glucosidase	+	−	+	+
*Antibiotic resistance (50 μg/mL)*				
Gentamicin	+	+	+	−
Kanamycin	+	−	−	−
Apramycin	−	−	+	+
Neomycin	−	−	+	−

1. s54d21^T^; 2. *H. minutum* DSM 14724^T^; 3. *H. gracilis* DSM 14753^T^; 4. *H. versicolor* H56D21^T^. All data were obtained from this study under the same conditions. +, positive; −, negative.

Hyalanone B (**2**): amorphous solid; [*α*]^25^_D_ − 20 (*c* 0.1, MeOH); UV (MeOH) *λ*_max_ nm (log *ε*): 232 (4.0), 326 (3.8); IR (NaCl) *ν*_max_ cm^−1^: 3364, 2,969, 2,922, 2,851, 1,649, 1,537, 1,468, 1,385, 1,335, 1,217, 1,077, 1,017, 900, 814, 643, and 574; ^1^H and ^13^C NMR data (CD_3_OD) (see Table 1); HRESIMS m/z 219.1105 [M + Na]^+^ (calcd for C_10_H_16_N_2_O_2_Na, 219.1104).

Hyalanone C (**3**): amorphous solid; [*α*]^25^_D_ − 11 (*c* 0.1, MeOH); UV (MeOH) *λ*_max_ nm (log *ε*): 232 (3.9), 318 (3.6); IR (NaCl) *ν*_max_ cm^−1^: 3363, 2,969, 2,923, 2,852, 1,650, 1,537, 1,468, 1,384, 1,359, 1,338, 1,217, 1,116, 1,088, 619, and 577; ^1^H and ^13^C NMR data (CD_3_OD) (see Table 1); HRESIMS *m/z* 233.1262 [M + Na]^+^ (calcd for C_11_H_18_N_2_O_2_Na, 233.1260).

#### 2.6.4 Theoretical computation method for structural identification

In general, conformational analyses were carried out via random searching in the Sybyl-X 2.0 (2013) using the MMFF94S force field with an energy cutoff of 5.0 kcal/mol. The results showed 15 lowest energy conformers. Subsequently, geometry optimizations and frequency analyses were implemented at the B3LYP-D3 (BJ)/6-31G* level in CPCM acetonitrile using ORCA5.0.1 (Neese, 2012, 2017). All conformers used for property calculations in this study were characterized to be stable point on potential energy surface (PES) with no imaginary frequencies. The excitation energies, oscillator strengths, and rotational strengths (velocity) of the first 60 excited states were calculated using the TD-DFT methodology at the PBE0/def2-TZVP level in CPCM acetonitrile using ORCA5.0.1. The ECD spectra were simulated by the overlapping Gaussian function (half the bandwidth at 1/e peak height, sigma = 0.30 for all) (Stephens and Harada, 2010). Gibbs free energies for conformers were determined by using thermal correction at B3LYP-D3 (BJ)/6-31G* level, and electronic energies were evaluated at the wB97M-V/def2-TZVP level in CPCM methanol using ORCA5.0.1. To get the final spectra, the simulated spectra of the conformers were averaged according to the Boltzmann distribution theory and their relative Gibbs free energy (∆G).

#### 2.6.5 Cytotoxicity assay

The cytotoxicity was determined by the MTS method as reported in our previous studies (Zang et al., 2020, 2022). Colorimetric assays were performed to evaluate the activity of each compound. A549 (human lung cancer cells), HepG-2 (human hepatocellular carcinoma cells), MCF-7 (human breast adenocarcinoma cells), RKO (human colon cells), and NCM460 (normal human colon mucosal epithelial cells) were purchased from the National Collection of Authenticated Cell Cultures of China. All cells were cultured in RPMI-1640 or DMEM medium (Hyclone, Logan, UT) supplemented with 10% fetal bovine serum (Hyclone) at 37°C in a humidified atmosphere with 5% CO_2_. Cell viability was assessed by conducting colorimetric measurements of the amount of insoluble formazan formed in living cells based on the reduction in 3-(4,5-dimethylthiazol-2-yl)-5(3-carboxymethoxyphenyl)-2-(4-sulfopheny)-2H-tetrazolium (MTS) (Sigma, St. Louis, MO). In brief, 100 μL of suspended adherent cells were seeded into each well of a 96-well cell culture plate and allowed to adhere for 12–24 h before the addition of a drug. In addition, suspended cells were seeded just before the addition of a drug, with an initial density of 1 × 10^5^ cells/ml in 100 μL of medium. Each cell line was exposed to each test compound at various concentrations in triplicate for 48 h. After the incubation, MTS solution (20 μL) was added to each well, and the incubation was continued for 2–4 h at 37°C. The optical density of the lysate was measured at 492 nm in a 96-well microtiter plate reader (MULTISKAN FC). The IC_50_ value of each compound was calculated by Reed and Muench’s method.

#### 2.6.6 Antimicrobial assay

The antimicrobial effect was determined following the method as reported in the previous study (Zang et al., 2016). Reference strains of *Staphylococcus aureus* (ATCC43300), *Escherichia coli* (ATCC11229), *Pseudomonas aeruginosa* (ATCC9027), and *Enterococcus faecalis* (ATCC29212) were cultivated for 24 h in Luria–Bertani medium (LB) at 37°C. Precultures of the tested microorganisms were made by inoculating 50 mL of LB medium and incubated for 24 h at 37°C. A culture suspension was made by 1/1000 dilution (OD600 0.01) from preculture and seeded in 96-well microtitration plates. In total, 1 μL of two-fold serial dilutions of each compound (40 mM) was prepared in 200 μL of medium. After 24 h, the optical density of the bacterial suspension of each well was measured at 595 nm using a multiplate reader (MULTISKAN FC). The MIC, which is the minimal concentration of a compound resulting in a 90% decrease in the number of microbial cultures compared with a control (DMSO only), was determined by curve fitting for bacteria. Vancomycin (Sigma–aldrich, Merck) was used as positive control.

#### 2.6.7 Anti-inflammatory assay

RAW 264.7 cells were obtained from National Collection of Authenticated Cell Cultures (Shanghai, China) and maintained in DMEM containing 10% fetal bovine serum (FBS) (Gibco BRL Co, Grand Island, NY, United States) at 37°C in humidified incubator containing 5% CO_2_. All tested compounds were dissolved in DMSO (the final concentration of DMSO was less than 0.25% in assay). Cell viability was evaluated by the CCK-8 method after 24 h of incubation with test compounds of 40 μM. RAW 264.7 cells were seeded into 96-well plates (2.5 × 10^5^ cells/well) for 24 h and then pretreated with test compounds. After being incubated for 3 h, the cells were stimulated with 100 ng/mL LPS (final concentration) for another 24 h. Celastrol (Sigma–aldrich, Merck) was used as the positive control. NO content in the supernatant was measured using Griess reagent. The absorbance at 540 nm was measured on a microplate reader (MULTISKAN FC).

## 3 Results and discussion

### 3.1 Phylogenetic analysis based on 16S rRNA gene sequence

**Figure 1:**
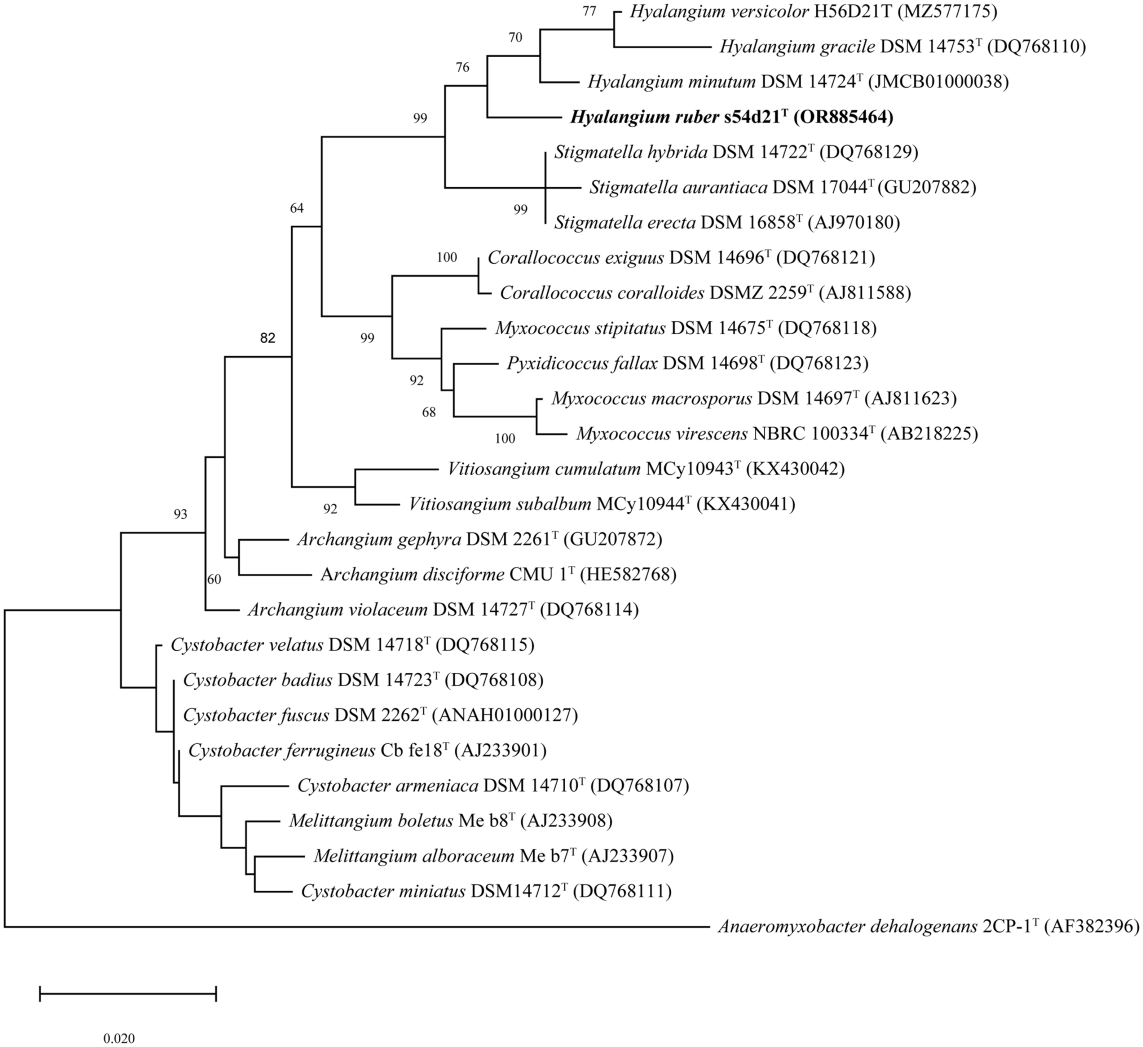
Maximum likelihood phylogenetic tree based on 16S rRNA gene sequences showing the relationships between strain s54d21^T^ and the closely related taxa. Bootstrap values were expressed as percentages of 1,000 replicates. More than 50% bootstrap values are shown. Bar, 0.02 the number of substitutions per site. The outgroup of the tree is *Anaeromyxobacter dehalogenans* 2CP-1^T^.

The nearly complete 16S rRNA gene sequence of the strain s54d21^T^ was obtained with the length of 1,536 bp (GenBank accession number: OR885464). Pairwise comparison of 16S rRNA gene revealed that the strain s54d21^T^ shared the highest similarity to *H. minutum* DSM 14724^T^ (98.2%), followed by *H. versicolor* H56D21^T^ (97.8%) and *H. gracilis* DSM 14753^T^ (97.7%). Phylogenetic analysis based on 16S rRNA gene sequence showed that the strain s54d21^T^ located in the genus *Hyalangium* and formed an individual branch containing all type strains of *Hyalangium* genus (Figure 1).

### 3.2 Genome sequencing and phylogenomic analysis

**Figure 2:**
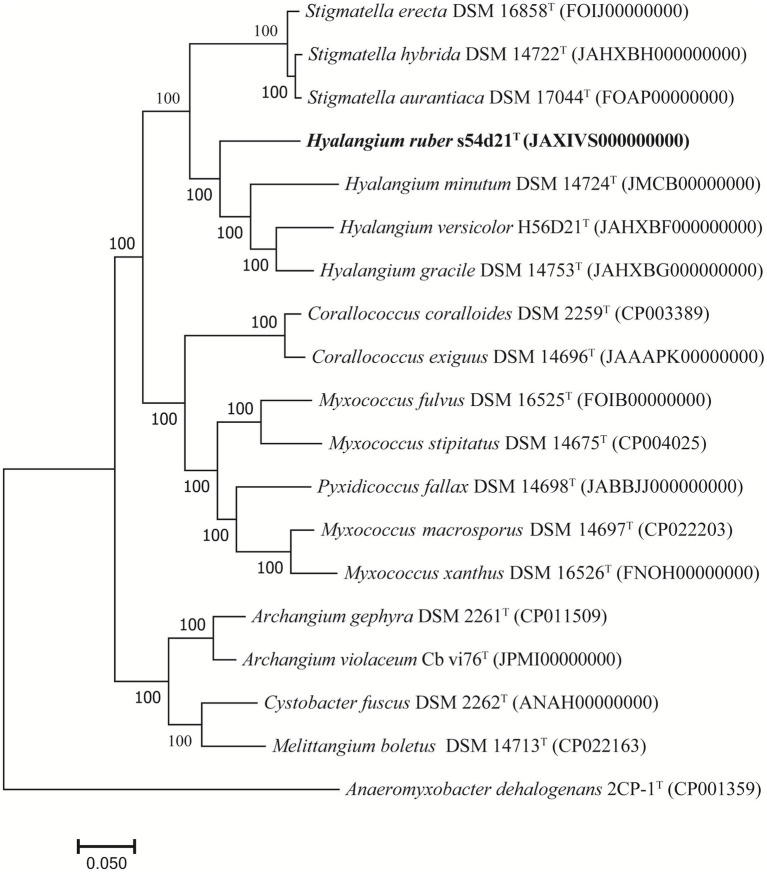
Phylogenomic tree reconstructed by the UBCG platform based on 92 core genes showing the relationships between strain s54d21^T^ and some closely related type strains. Bar, 0.05 represents the number of substitutions per site.

The draft genome of the strain s54d21^T^ was 10.77 Mb in size with 43 contigs, the N50 of 586,569 bp, and the DNA G + C content of 68.5 mol% (Supplementary Table S1). The genome contained 8,621 protein-coding sequences, 70 tRNAs, and 3 rRNAs genes. The UBCG-based phylogenomic tree showed that the strain s54d21^T^ was closely related to all type species of *Hyalangium* (Figure 2), which was consistent with the tree topology based on the 16S rRNA genes. The calculated genome similarity values between the strain s54d21^T^ and other type species in the genus *Hyalangium* were in the ranges of 81.3–82.7% for ANI and 24.4–25.7% for dDDH (Supplementary Table S2), which were all below the threshold values for species delineation (95.0–96.0% for ANI and 70.0% for dDDH) (Goris et al., 2007; Richter and Rosselló-Móra, 2009). These results suggested that the strain s54d21^T^ should be considered as a novel species in the genus *Hyalangium*, for which the name *Hyalangium ruber* sp. nov was proposed.

The RAST annotation result showed that almost 81.0% of coding sequences in the strain s54d21^T^ were not assigned to subsystems. The general feature of subsystem category distribution in the strain s54d21^T^ genome was similar to those in its closest phylogenetic neighbor. No genes involved in motility and chemotaxis were found to be encoded by the strain s54d21^T^ genome and in the genomes of closely related type strains. However, genes associated with iron acquisition and metabolism were found in the strain s54d21^T^ more than these in the three closely related strain genomes (Supplementary Figure S1). Based on CAZy database annotation, the genome of the strain s54d21^T^ contained 23 carbohydrate-binding modules, 33 carbohydrate esterases, 78 glycoside hydrolases, 61 glycosyl transferases, 6 polysaccharide lyases, and 15 auxiliary activities (Supplementary Figure S1). There were most types of carbohydrate enzymes involved in glycoside hydrolases, which can hydrolyze polysaccharides such as starch and peptidoglycan. A total of 32 biosynthetic gene clusters (BGCs) were identified in the strain s54d21^T^ genome, including three NRPS-like fragments, a PKS-type cluster, four hybrid PKS/NRPS-type cluster, nine RiPP-type clusters, five terpenes-type clusters, two lanthipeptides-type clusters, and others (Supplementary Figure S1).

### 3.3 Morphological, physiological, and biochemical characteristics

**Figure 3:**
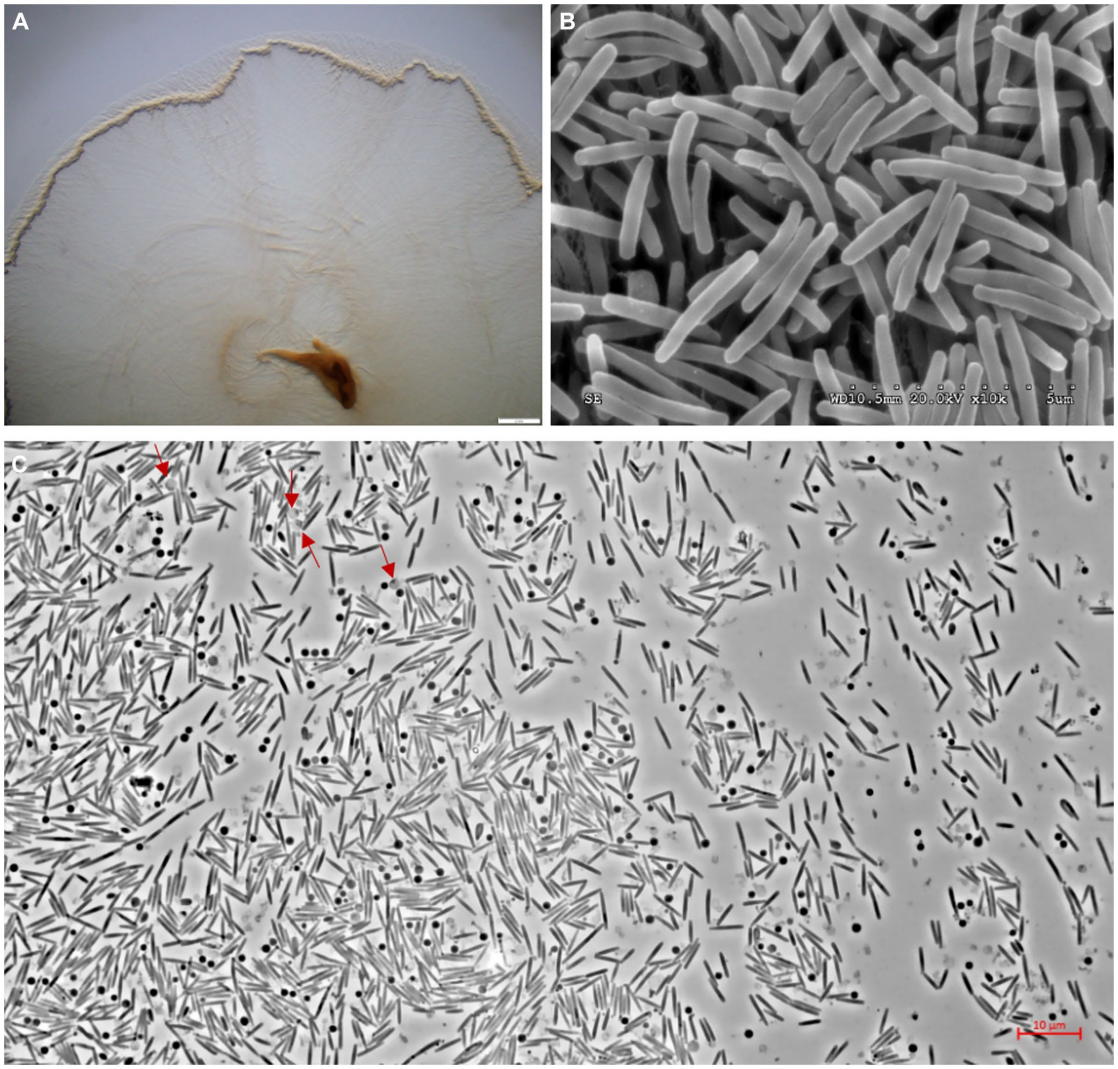
Photographs showing colonies and cell morphology of the strain s54d21^T^. **(A)** The stereoscopic photographs of swarming colonies on VY/2 agar after 5 days of incubation at 30°C (bar, 2 mm). **(B)** The transmission electron microscopic photographs of vegetative cells (bar, 5 μm). **(C)** The phase contract microscopic photograph of rod-shaped vegetative cells, spherical sporangioles (red arrow), and spherical myxospores (bar, 10 μm).

The swarms of the strain s54d21^T^ exhibited circular, red, thin film with wavy flared edges on VY/2 agar (Figure 3A). Vegetative cells were slender rods with tapered ends measuring 0.5–0.7 × 3–7 μm (Figure 3B). Sporangioles were spherical, some of which were empty and transparent (Figure 3C). Myxospores were irregular spherical (Figure 3C). Strain s54d21^T^ showed positive to oxidase, catalase, starch, aesculin hydrolysis, gelatin hydrolysis, alkaline phosphatase, esterase (C4), esterase lipase (C8), lipase (C14), leucine arylamidase, valine arylamidase, acid phosphatase, naphtol-AS-Bl-phosphoamidase, *β*-glucosidase, and *N-*acetyl*-β*-glucosaminidase. The differentiating characteristics with other type strains in the genus *Hyalangium* are shown in Table 1. Strain s54d21^T^ could be distinguished from its closely related type strains in temperature for growth, *β-*galactosidase, cystine arylamidase, trypsin, *α*-chymotrypsin, *β*-glucosidase and several antibiotic sensitivities.

The major respiratory quinone detected in the strain s54d21^T^ was menaquinone-8 (MK-8). The major fatty acids (>5% of the total amounts) contained iso-C_17:0_ 2-OH (27.8%), iso-C_15:0_ (17.4%), iso-C_15:0_ DMA (8.2%), iso-C_16:0_ (6.8%), C_16:1_*ω*5c (6.6%), and iso-C_15:0_ 3-OH (6.2%) (Supplementary Table S3). The high levels of iso-C_15:0,_ C_16:1_*ω*5c, and iso-C_15:0_ DMA were found in all type strains in the genus *Hyalangium* and seemed to be the major fatty acid in this genus. Moreover, the remarkably high amount of iso-C_17:0_ 2-OH and the low proportions of C_16:1_*ω*5c, C_16:0,_ C_16:1_*ω*7c, and iso-C_17:0_ in the strain s54d21^T^ could distinguish the strain s54d21^T^ form its neighbor-type species in the genus *Hyalangium*.

### 3.4 Description of *Hyalangium ruber* sp. nov

*Hyalangium ruber* (ru′ber. L. masc. Adj. ruber red, referring to the color of the colonies).

Cells are gram-negative. Growth occurred at 20–40°C, pH 4.0–10.0 with the NaCl tolerance of 0–0.5% (m/v). Resistant to ampicillin, gentamicin, kanamycin, polymyxin B, and bacitracin B, sensitive to erythromycin, nalidixic acid, rifampin, spectinomycin, streptomycin, tetracycline, apramycin, chloramphenicol, neomycin, trimethoprim, and oxytetracycline. Major cellular fatty acids are iso-C_17:0_ 2-OH, iso-C_15:0_, iso-C_15:0_ DMA, iso-C_16:0_, C_16:1_*ω*5c, and iso-C_15:0_ 3-OH. Predominant respiratory quinone is MK-8. The genomic DNA G + C content of the type strain is 68.5 mol%.

The type strain s54d21^T^ (= GDMCC 1.1945^T^ = JCM 39263^T^) was isolated from the sediment sampled from Xinghu National Wetland Park located in Guangdong Province, China. The GenBank accession numbers for the 16S rRNA gene and whole genome sequences of the type strain are OR885464 and JAXIVS000000000, respectively.

### 3.5 Structure elucidation of new compounds

**Figure 4:**
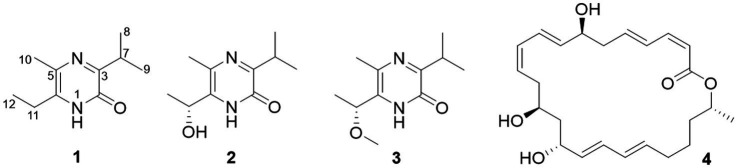
Chemical structures of isolated compounds (1–4).

The gene sequence analyses of the strain s54d21^T^ supposed the production of polyketides and peptides in terms of the presence of PKS and NRPS BGCs. Therefore, the chemical investigation of the culture broth of the strain was performed as described. Here, the structure including absolute configurations of three new compounds (**1**–**3**) was elucidated (Figure 4).

**Figure 5:**
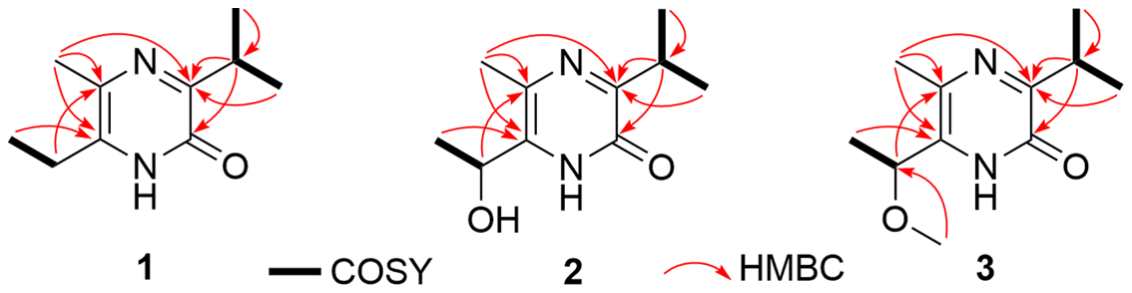
Key ^1^H-^1^H COSY and HMBC correlations of compounds (1–3).

Compound **1** was obtained as an amorphous solid, and the molecular formula of C_10_H_16_N_2_O was determined by the positive mode HRESIMS with a [M + Na]^+^ ion peak at *m/z* 203.1150, indicating four degrees of unsaturation. The 1D NMR data (Table 2) and HSQC spectrum presented signals of ten carbons, including four methyls (a singlet at *δ*_H_ 2.22, two doublets at *δ*_H_ 1.20, and a triplet at *δ*_H_ 1.17), a methylene at *δ*_H_ 2.57 (q, *J* = 7.5 Hz) and *δ*_C_ 26.1, a methine at *δ*_H_ 3.34 (m) and *δ*_C_ 30.9, and four unprotonated carbons. ^1^H–^1^H COSY correlations demonstrated the existence of two spin systems of an ethyl group and an isopropyl moiety (Figure 5). Combination analyses of the chemical formula and HMBC correlations (Figure 5) revealed that four unprotonated carbons (C-2, C-3, C-5, and C-6) and two nitrogens were assigned to the 2(1*H*)-pyrazinone ring. The comparison of those spectroscopic data suggested that structure of **1** was such close to those knowns featuring a 3,5,6-substitued 2(1*H*)-pyrazinone core structure (Motohashi et al., 2011; Ma et al., 2020). The differences between **1** and known compounds were three substituent groups located at C-3, C-6, and C-5, including an abovementioned ethyl group, an isopropyl group, and a methyl (Me-10), respectively. Subsequent analyses of HMBC spectrum supported those assignments for structure **1**, where key HMBC correlations from H-7/Me-8 to C-2, H-7/Me-8/Me-10 to C-3, Me-10/H-11 to C-5, and Me-10/Me-12 to C-6 were observed (Figure 5). Hence, compound **1** was unambiguously determined as a 3,5,6-trisubstituted 2(1*H*)-pyrazinone and designated as hyalanone A.

**Table 2:** ^1^H (150 MHz) and ^13^C (600 MHz) NMR data of compounds **1**–**3** recorded in CD_3_OD (*δ* in ppm; *J* in Hz).

Pos.	**1**		**2**		**3**	
	*δ*_H_	*δ*_C_	*δ*_H_	*δ*_C_	*δ*_H_	*δ*_C_
2	–	157.3	–	157.5	–	157.4
3	–	160.9	–	161.0	–	161.5
5	–	131.3	–	132.8	–	132.8
6	–	135.8	–	135.3	–	134.2
7	3.34 m	30.9	3.33 m	31.5	3.33 m	30.9
8	1.20 d 6.8	20.5	1.22 d 6.8	20.5	1.20 d 6.8	20.4
9	1.20 d 6.8	20.5	1.22 d 6.8	20.5	1.20 d 6.8	20.5
10	2.22 s	15.2	2.29 s	14.8	2.29 s	14.8
11	2.57 q 7.5	26.1	4.89 q 6.5	66.6	4.52 q 6.5	76.6
12	1.17 t 7.5	13.7	1.44 d 6.5	22.5	1.47 t 6.5	19.5
11-OCH_3_	–	–	–	–	3.22 s	55.9

**Figure 6:**
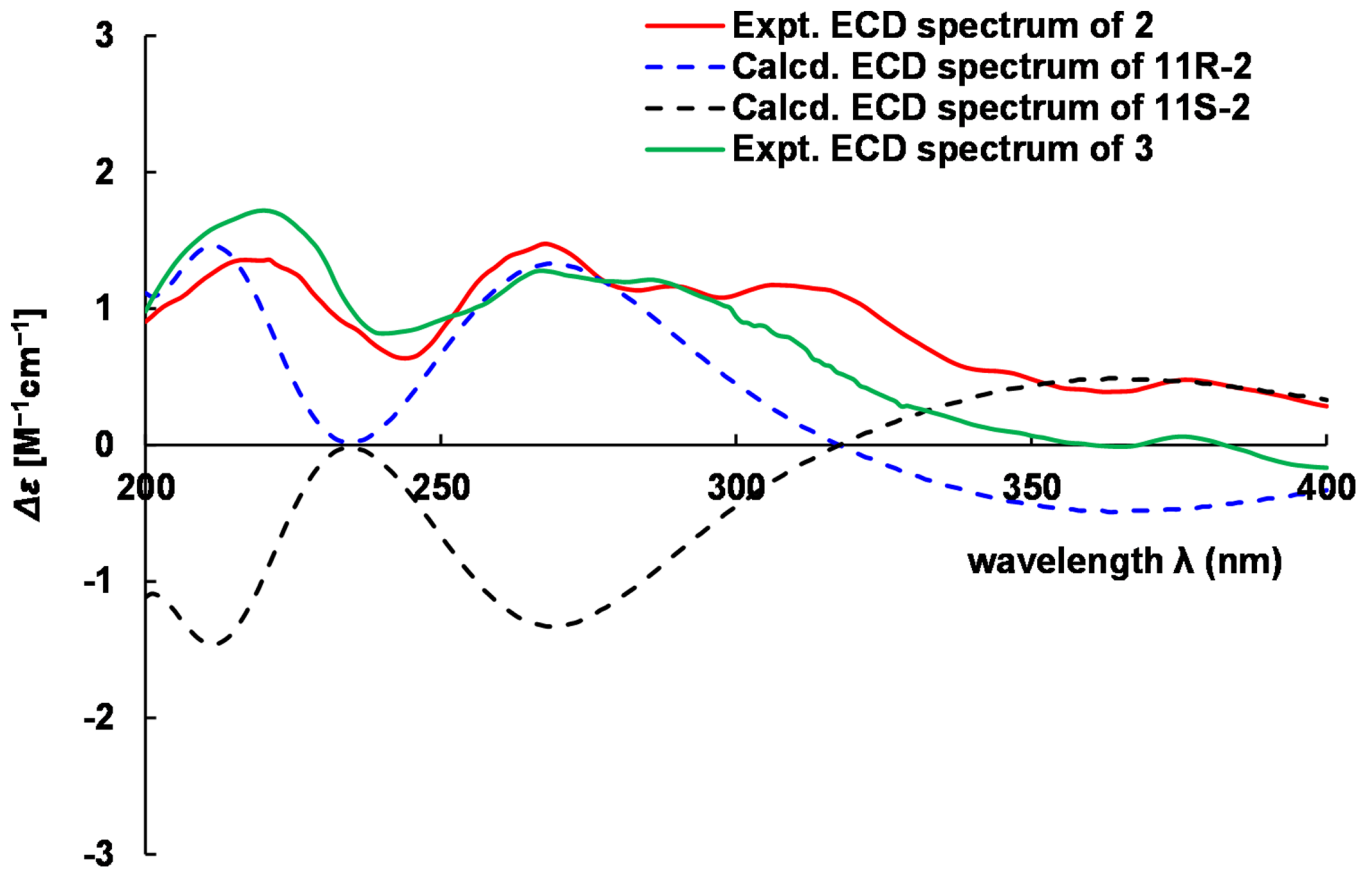
Comparison of experimental and calculated ECD of 2 and 3.

Hyalanone B (**2**) was also isolated as an amorphous solid. The molecular formula was demonstrated to be C_10_H_16_N_2_O_2_ via a positive HRESIMS ion peak at *m/z* 219.1105 [M + Na]^+^. The ^1^H and ^13^C NMR data of **2** was such close to those of **1,** except for an oxygenated methine at *δ*_H_ 4.89 (q, *J* = 6.5 Hz, H-11) and *δ*_C_ 66.6 (C-11) (Table 2), suggesting a similar 2(1*H*)-pyrazinone skeleton. A hydroxyethyl group replaced the ethyl at C-6 of **1**, which was further confirmed by H–^1^H COSY cross-peaks and key HMBC correlations from Me-12 to C-6/C-11 and H-11 to C-5 (Figure 5). There is not enough compound **2** for Mosher’s method to determine the absolute configuration of the chiral center C-11. Therefore, it was inferred from theoretical ECD calculation by using TDDFT methodology at PBE0/def2-TZVP level in CPCM acetonitrile using ORCA5.0.1 (Neese, 2012, 2017). As shown in Figure 6, the experimental ECD curve of **2** was well matched with the calculated one that concluded the absolute configuration of C-11 to be *R*.

The molecular formula C_11_H_18_N_2_O_2_ of **3** was deduced from a HRESIMS spectrum. Analyses of 1D NMR data (Table 2) and HMBC correlations (Figure 5) of **3** exhibited a highly similarity with those of **2**, where the unique difference between them was an extra methoxyl at *δ*_H_ 3.22 and *δ*_C_ 55.9 (11-OMe) in **3**. Moreover, key HMBC correlation from 11-OMe to C-11 indicated the location of a methoxyl at C-11 of **3** rather than a hydroxyl in **2** (Figure 5). By comparing of experimental ECD curves of **2** and **3** (Figure 6), the absolute configuration of C-11 was still approved to be *R*. Therefore, compound **3** was established and given the name hyalanone C.

2(1*H*)-Pyrazinones are a type of natural products originally biosynthesized by NRPS gene cluster with two amino acids (Wyatt et al., 2010; Zimmermann and Fischbach, 2010; Wyatt et al., 2012). Based on biosynthetic pathways of pyrazinone analogs, three new compounds are proposed to be derived from the valine and alanine or threonine building blocks. Analyses of the genome sequence of strain s54d21^T^ by antiSMASH showed three NRPS-like and three PKS-NRPS BGCs. Among them, the NRPS (Region 1.3) and PKS-NRPS (Region 5.2) gene clusters containing a *Val* module are potent BGCs related to the biosynthesis of those new pyrazinones (Supplementary Figure S2).

### 3.6 Biological assays

The previous report of pyrazinone derivatives described their remarkable cytotoxicity against human cancer cell lines and antimicrobial activity (Jansen et al., 2014). Therefore, cytotoxic activities against human cancer cell lines and antimicrobial and anti-inflammatory effects of all isolates were tested in this study. The cytotoxicity evaluation of compounds **1**–**4** was performed using the MTS method, where taxol was used as the positive control. Among them, compound **4** displayed moderate cytotoxicity against A549, RKO, and NCM460 with IC_50_ values at 32.14 ± 2.25, 27.31 ± 8.96, and 31.33 ± 1.71 μM (positive control with IC_50_ values <0.008 μM), respectively. In addition, their inhibition of NO production in LPS-induced RAW 264.7 cells was tested. All compounds showed weak inhibitory effect (< 10.0%) of NO production at the concentration of 40 μM. The evaluation of their antimicrobial activities against *S. aureus*, *E. coli*, *P. aeruginosa*, and *E. faecalis* did not show obvious inhibition at the concentration of 200 μM.

Pyrazinones are previously reported to regulate phenotypic changes in the pathogen (e.g., quorum sensing) or act directly on the host (e.g., virulence factors), such as tyrvalin and phevalin are used as regulators of virulence factor gene expression (Wyatt et al., 2010; Secor et al., 2012). Thus, although three new pyrazinones do not exhibit obvious effects in the evaluation of cytotoxic, antimicrobial, and anti-inflammatory activities, the discovery of new molecules provides potential for further research studies of relationships between the small molecule and microorganisms as well as the role of myxobacteria in nature.

## 4 Conclusion

In this study, a novel myxobacterium s54d21^T^ belonging to a rare *Hyalangium* genus, designated *Hyalangium ruber* sp. nov, was purified from a wetland sediment using the *E. coli* baiting method. Uncovered by analysis of the gene sequence, the further chemical investigation of bioactive secondary metabolites from culture broth of *H. ruber* led to the production of three unprecedented 3,5,6-trisubstituted 2(1*H*)-pyrazinone derivatives, namely, hyalanones A–C (**1**–**3**), together with a known macrolactin A (**4**). In biological assay, compound **4** exhibited modest cytotoxic activities against human cell lines A549, RKO, and NCM460 with IC_50_ values between 27.31 and 32.14 μM. Although three new compounds have not exhibited significant activities in biological assays, their structures enrich the chemical diversity of 2(1*H*)-pyrazinones from myxobacteria.

## Data availability statement

The datasets presented in this study can be found in online repositories. The names of the repository/repositories and accession number(s) can be found at: NCBI, OR885464 and JAXIVS000000000.

## Author contributions

YZa: Supervision, Writing – review & editing, Writing – original draft, Project administration, Funding acquisition, Formal analysis, Data curation, Conceptualization. XZ: Writing – original draft, Methodology, Funding acquisition, Formal analysis, Data curation, Conceptualization. ZW: Writing – original draft, Methodology, Investigation, Data curation. QT: Writing – review & editing, Methodology. YZh: Writing – review & editing, Conceptualization. QY: Writing – review & editing, Supervision, Conceptualization. HZ: Writing – review & editing, Supervision, Funding acquisition, Conceptualization.
