# Supplementary material for: Hyalangiumruber sp. nov, characterization of a novel myxobacterium strain s54d21 and their secondary metabolites
Source: Front Microbiol. 2024 Mar 8;15:1369499. doi: 10.3389/fmicb.2024.1369499 (PMC10959286; doi:10.3389/fmicb.2024.1369499)
Supplement: Supplementary file 1 [file Data_Sheet_1.pdf]

Supplementary Material

Hyalangium ruber sp. nov, characterization of a novel myxobacterium strain s54d21 and their secondary metabolites

Yi Zang[1†], Xianjiao Zhang[1,2†], Zhe Wang[1], Qingyi Tong[3], Yang Zhou[1], Qing Yao[2]*, Honghui Zhu[1]*

[1] Key Laboratory of Agricultural Microbiomics and Precision Application (MARA), Guangdong Provincial Key Laboratory of Microbial Culture Collection and Application, Key Laboratory of Agricultural Microbiome (MARA), State Key Laboratory of Applied Microbiology Southern China, Institute of Microbiology, Guangdong Academy of Sciences, Guangzhou, P.R. China

[2] College of Horticulture, South China Agriculture University, Guangzhou, P.R. China

[3] School of Pharmacy, Tongji Medical College, Huazhong University of Science and Technology, Wuhan, P.R. China

*** Correspondence:**
Honghui Zhu

zhuhh_gdim@163.com

Qing Yao

yaoqscau@scau.edu.cn

Table of Contents

Table S1. Genomic features of strain s54d21^T and the closely type strains. 3
Table S2. ANI and dDDH values between strain s54d21^T and the closely type strains. 3
Table S3. Cellular fatty acid profiles of strain s54d21^T and the closely related type strains. 3
Figure S1. Distribution of genes in strain s54d21^T and its closely related type strains. (A) RAST annotation; (B) dbCAN annotation; (C) antiSMASH annotation. 4
Figure S2. The NRPS-like (Region 1.3) and PKS-NRPS (Region 5.2) gene clusters within strain s54d21^T. 5
Figure S3. ^{1}H NMR spectrum of **1** in methanol-*d*4. 6
Figure S4. ^{13}C NMR spectrum of **1** in methanol-*d*4. 6
Figure S5. DEPT-135° spectrum of **1** in methanol-*d*4. 7
Figure S6. HSQC spectrum of **1** in methanol-*d*4. 7
Figure S7. ^{1}H–^{1}H COSY spectrum of **1** in methanol-*d*4. 8
Figure S8. HMBC spectrum of **1** in methanol-*d*4. 8
Figure S9. NOESY spectrum of **1** in methanol-*d*4. 9
Figure S10. (+) HRESIMS spectrum of **1**. 9
Figure S11. IR spectrum of **1**. 10
Figure S12. ^{1}H NMR spectrum of **2** in methanol-*d*4. 10
Figure S13. ^{13}C NMR spectrum of **2** in methanol-*d*4. 11
Figure S14. DEPT-135° spectrum of **2** in methanol-*d*4. 11
Figure S15. HSQC spectrum of **2** in methanol-*d*4. 12
Figure S16. ^{1}H–^{1}H COSY spectrum of **2** in methanol-*d*4. 12
Figure S17. HMBC spectrum of **2** in methanol-*d*4. 13
Figure S18. NOESY spectrum of **2** in methanol-*d*4. 13
Figure S19. (+)-HRESIMS spectrum of **2**. 14
Figure S20. IR spectrum of **2**. 14
Figure S21. ^{1}H NMR spectrum of **3** in methanol-*d*4. 15
Figure S22. ^{13}C NMR spectrum of **3** in methanol-*d*4. 15
Figure S23. DEPT-135° spectrum of **3** in methanol-*d*4. 16
Figure S24. HSQC spectrum of **3** in methanol-*d*4. 16
Figure S25. ^{1}H–^{1}H COSY spectrum of **3** in methanol-*d*4. 17
Figure S26. HMBC spectrum of **3** in methanol-*d*4. 17
Figure S27. NOESY spectrum of **3** in methanol-*d*4. 18
Figure S28. (+)-HR-ESI-MS spectrum of **3**. 18
Figure S29. IR spectrum of **3**. 19
Details of theoretical computation for compound 2 19

Table S1. Genomic features of strain s54d21^T and the closely type strains.

**Genomic features**	s54d21^T	*H. minutum* DSM 14724^T	*H. gracilis* DSM 14753^T	*H. versicolor* H56D21^T
Size (Mbp)	10.77	11.19	11.73	13.56
Number of contigs	43	44	82	101
DNA G+C content (mol%)	68.5	68.0	69.5	67.1
N50 (bp)	586569	504291	346585	364296
L50	7	7	11	12
Number of genes	8727	8967	9333	10609
Number of CDSs	8621	8882	9176	10522
Number of rRNAs genes	3	3	3	3
Number of tRNAs genes	70	81	89	83
Completeness (%)	99.76%	98.94%	98.94%	99.57%
GenBank accession number	JAXIVS000000000	JMCB00000000	JAHXBG000000000	JAHXBF000000000

Table S2. ANI and dDDH values between strain s54d21^T and the closely type strains.

Type strain	ANI values (%)	dDDH values (%)
H. minutum DSM 14724^T	81.7	24.5
H. gracilis DSM 14753^T	82.7	25.7
H. versicolor H56D21^T	81.3	24.4

Table S3. Cellular fatty acid profiles of strain s54d21^T and the closely related type strains.

**Fatty acids**	1	2	3	4
$C_{16:0}$	2.8	7.8	8.8	6.9
$C_{16:1}\omega5c$	6.6	22.1	24.3	14.3
$C_{16:1}\omega7c$	1.5	7.1	6.6	3.7
$C_{18:1}\omega9c$	−	1.3	0.4	1.1
$C_{16:0}$-2OH	−	1.5	−	0.9
iso-$C_{15:0}$	17.4	11.7	19.2	27.9
iso-$C_{16:0}$	6.8	10.7	1.5	3.3
iso-$C_{17:0}$	3.7	6.0	17.9	11.3
iso-$C_{17:1}\omega5c$	0.9	1.1	1.4	0.5
iso-$C_{15:0}$-3 OH	6.2	8.1	2.6	3.8
iso-$C_{17:0}$-2 OH	27.8	8.0	1.0	3.6
iso-$C_{15:0}$ OAG	0.5	1.7	0.4	0.6
iso-$C_{15:0}$ DMA	8.2	7.3	11.2	18.4

1. s54d21^T; 2. *H. minutum* DSM 14724^T; 3. *H. gracilis* DSM 14753^T; 4. *H. versicolor* H56D21^T. All data are obtained from this study. Values are percentages of total fatty acids detected. Fatty acids percentages less than 1% of the total amounts in all the strains are not shown. Components in bold represent major fatty acid profiles. −, not detected.

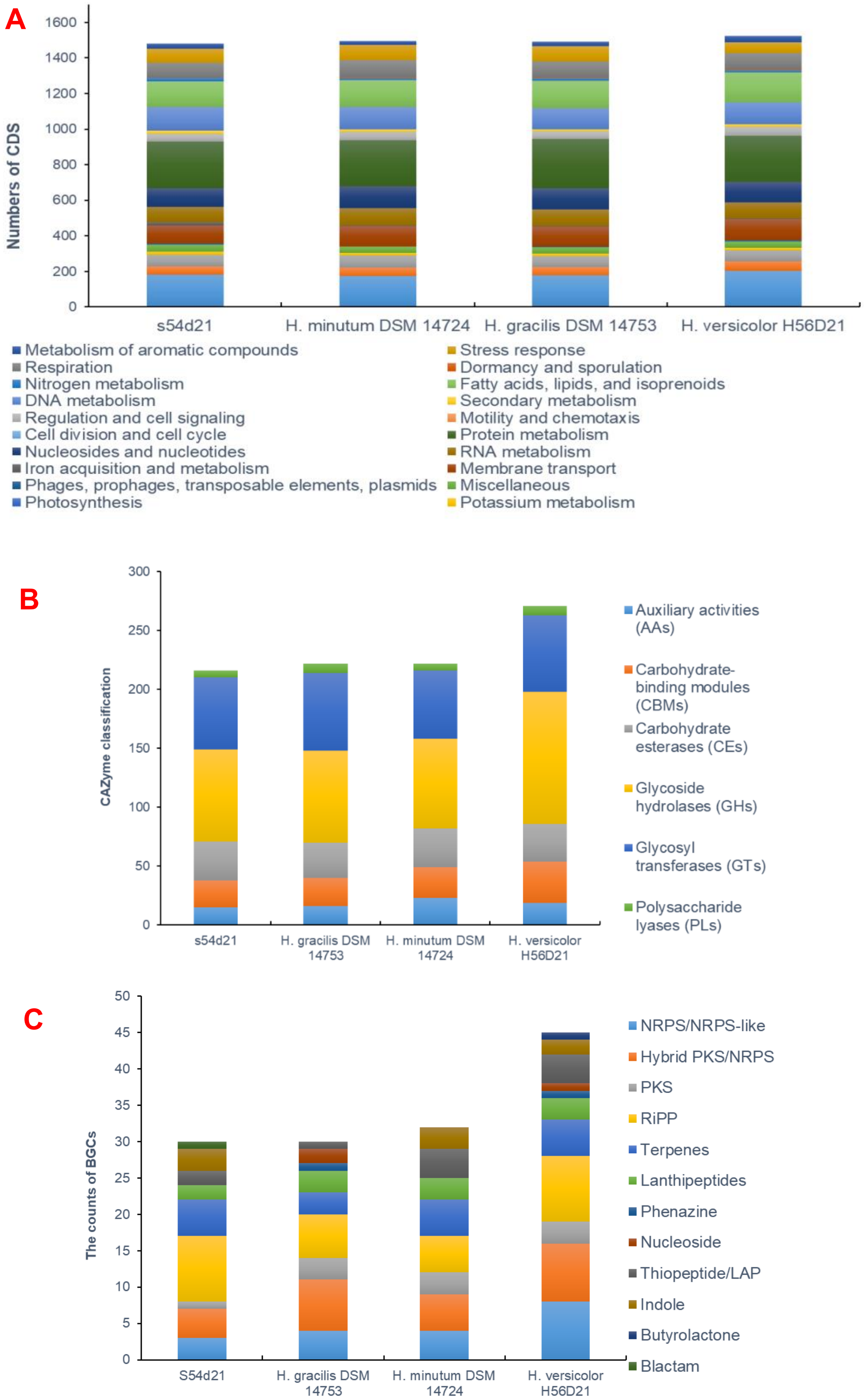

Figure S1. Distribution of genes in strain s54d21^{T} and its closely related type strains. (A) RAST annotation; (B) dbCAN annotation; (C) antiSMASH annotation.

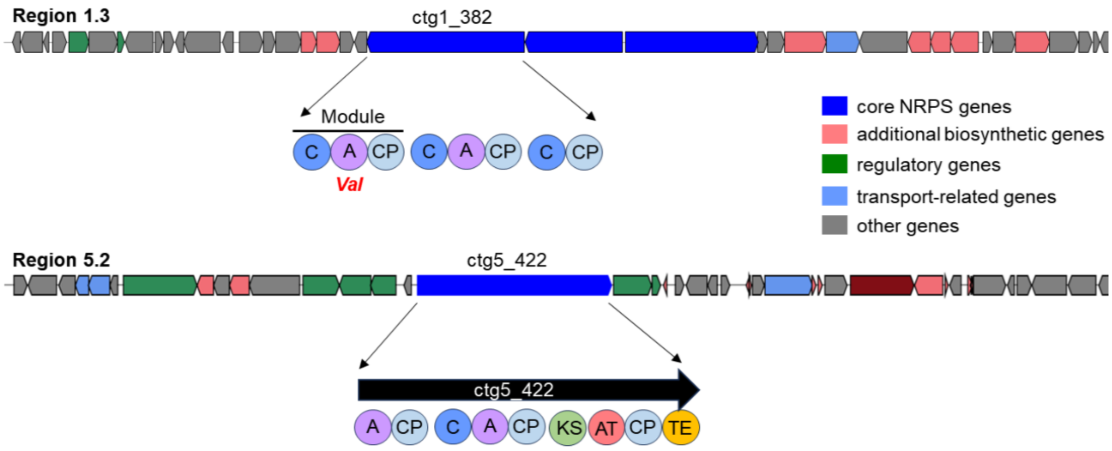

Figure S2. The NRPS-like (Region 1.3) and PKS-NRPS (Region 5.2) gene clusters within strain s54d21^{T}. The analyses of the gene cluster on the website of antiSMASH predicted the gene containing a *Val* module that were potent BGCs involved in the biosynthesis of those new pyrazinones.

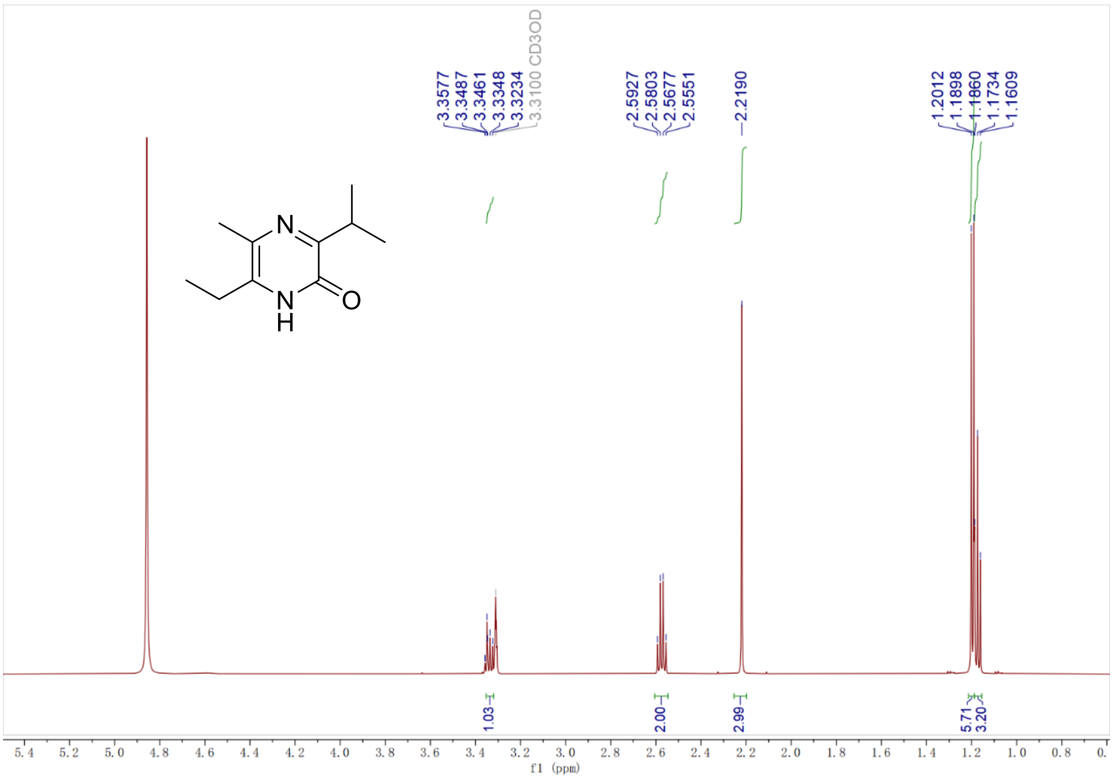

Figure S3. ^{1}H NMR spectrum of **1** in methanol-d_4.

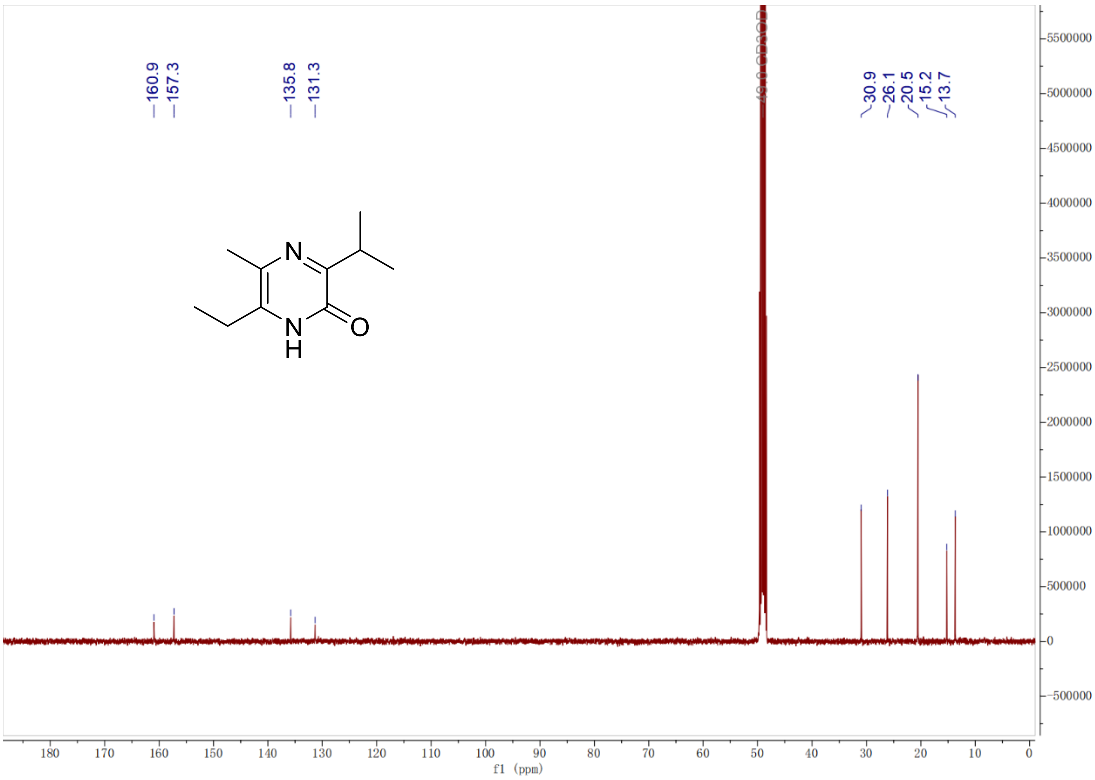

Figure S4. ^{13}C NMR spectrum of **1** in methanol-d_4.

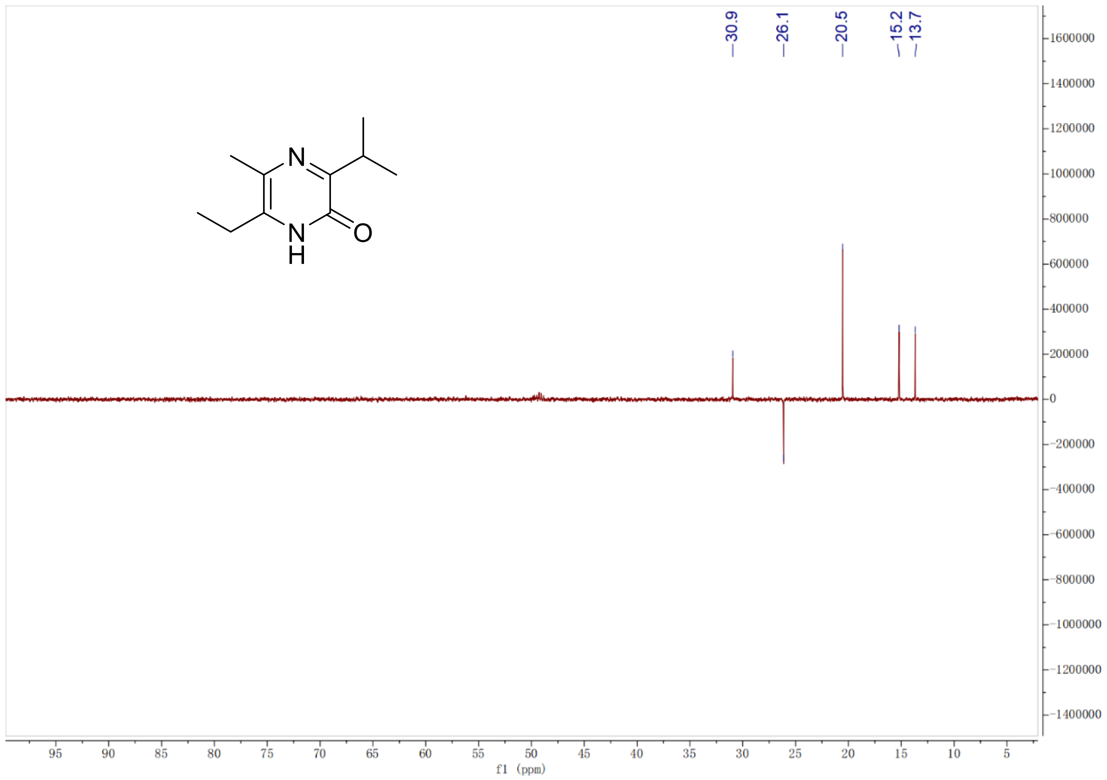

Figure S5. DEPT-135° spectrum of **1** in methanol-d_4.

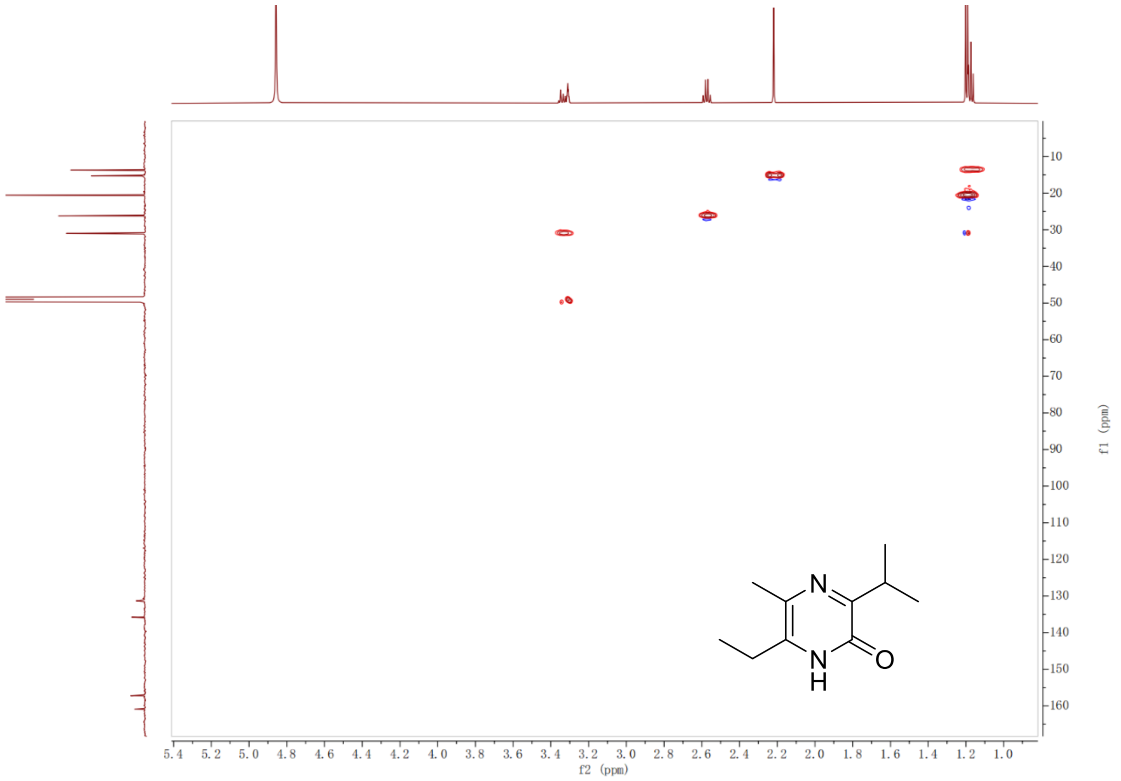

Figure S6. HSQC spectrum of **1** in methanol-d_4.

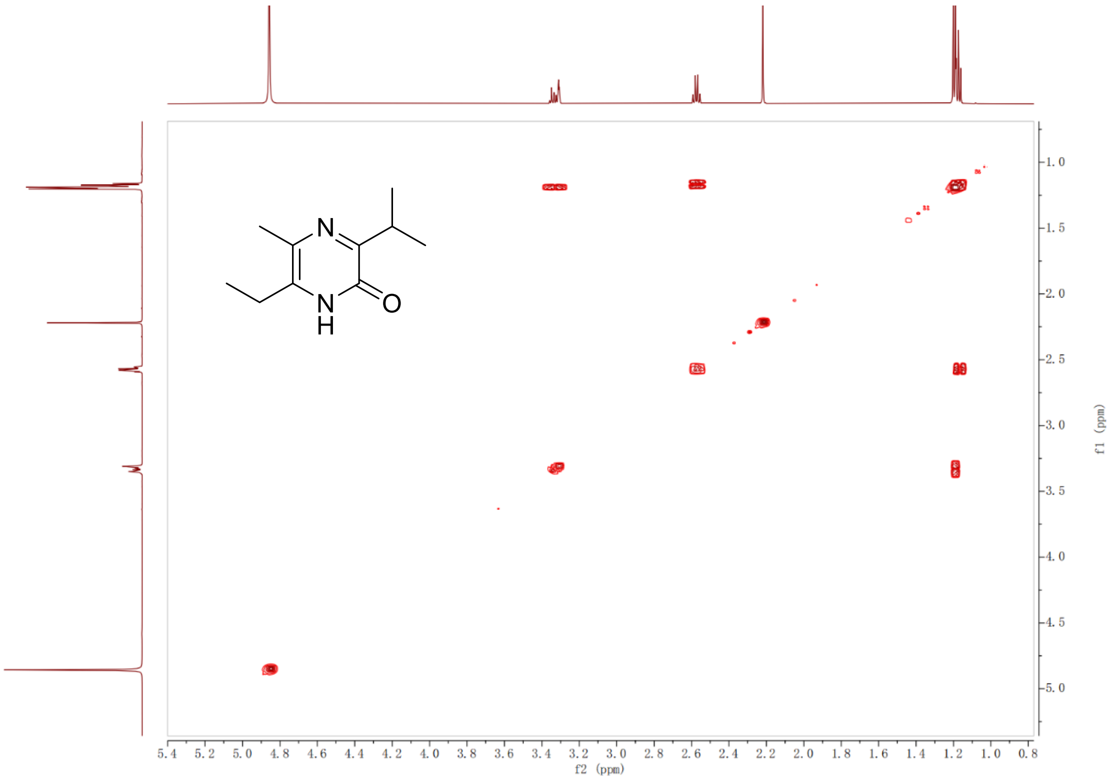

Figure S7. ^{1}H–^{1}H COSY spectrum of **1** in methanol-d_4.

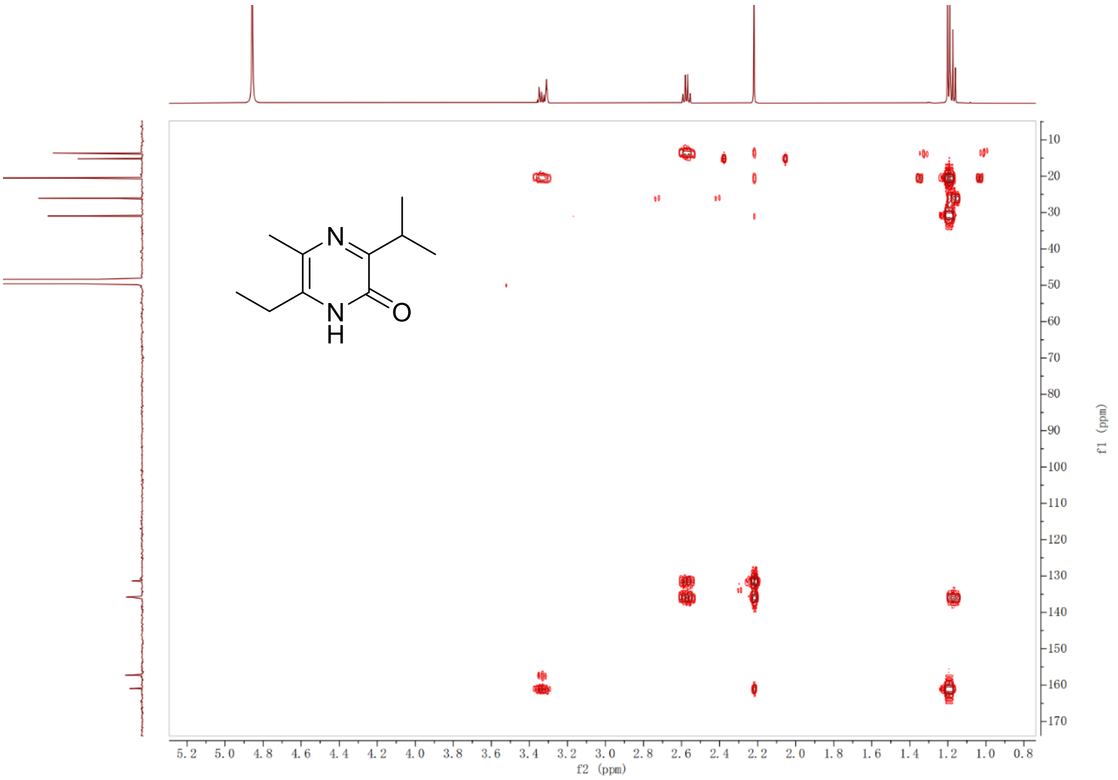

Figure S8. HMBC spectrum of **1** in methanol-d_4.

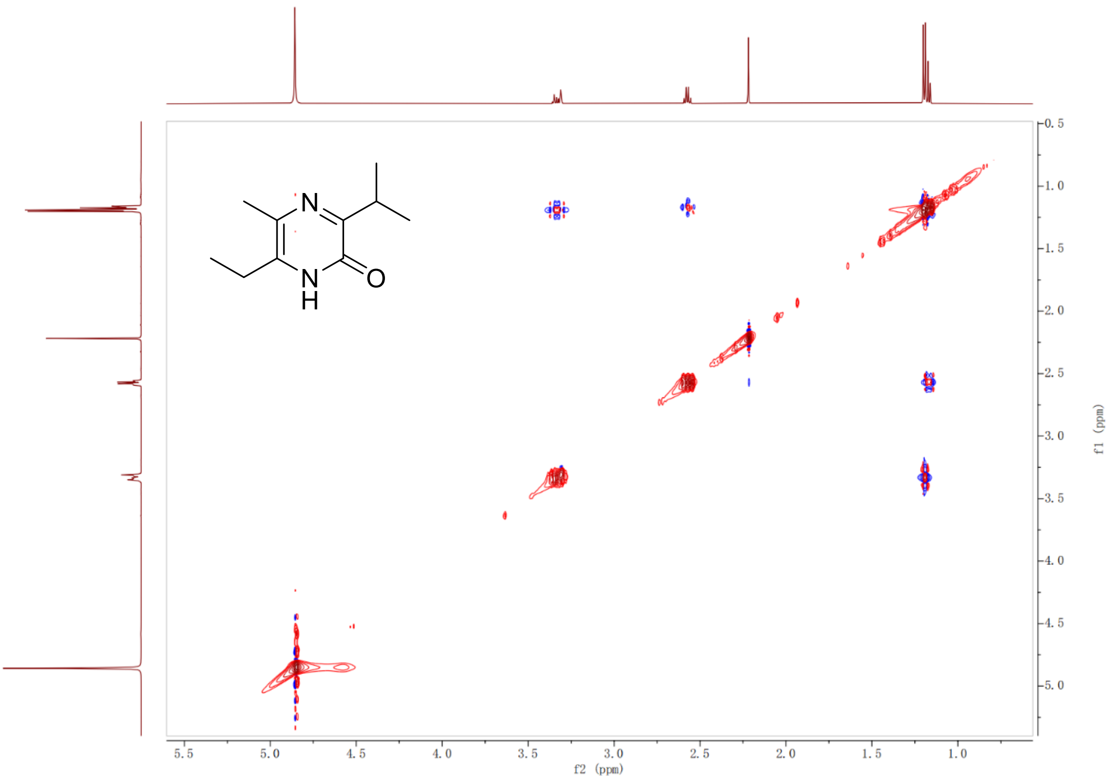

Figure S9. NOESY spectrum of **1** in methanol-d_4.

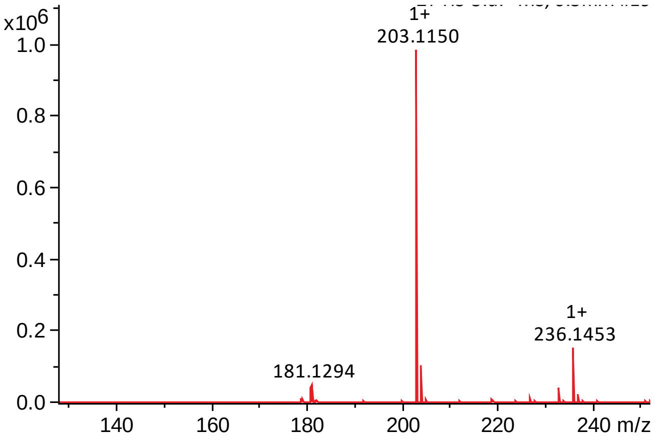

Figure S10. (+) HRESIMS spectrum of **1**.

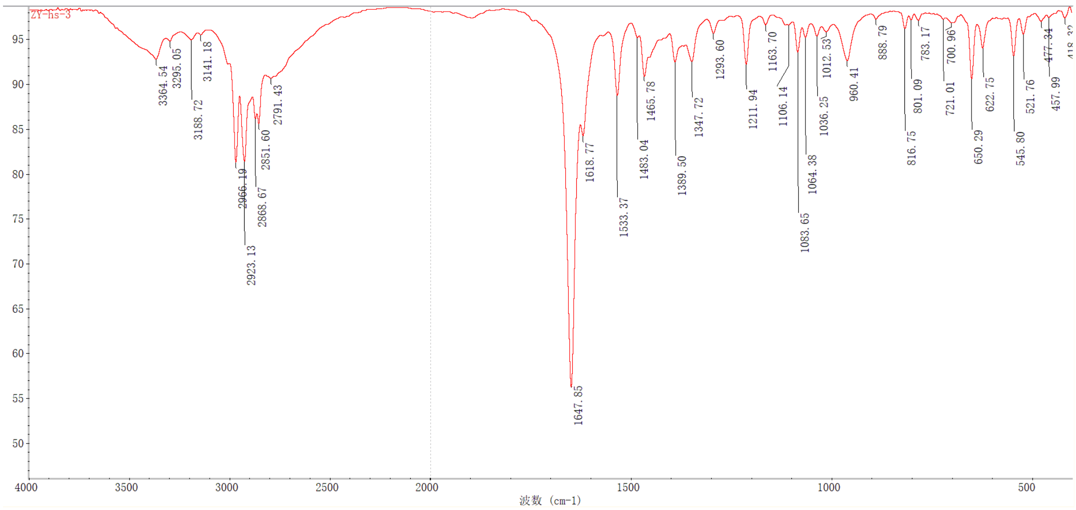

Figure S11. IR spectrum of **1**.

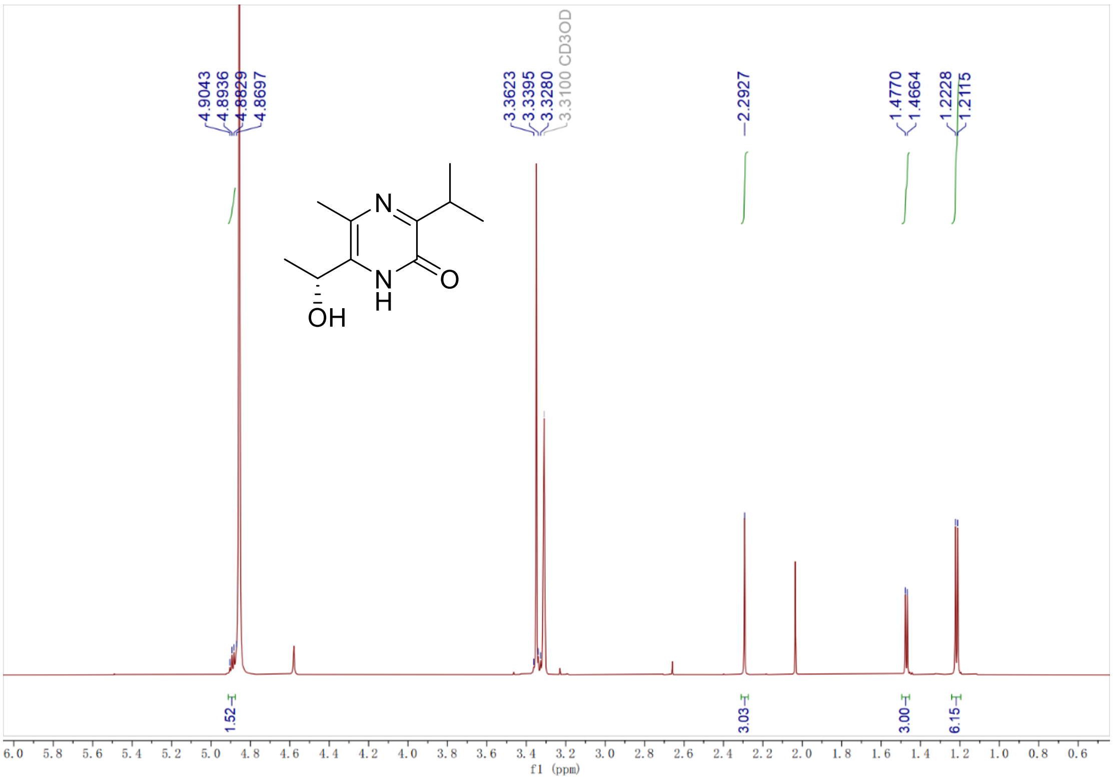

Figure S12. ^{1}H NMR spectrum of **2** in methanol-d_4.

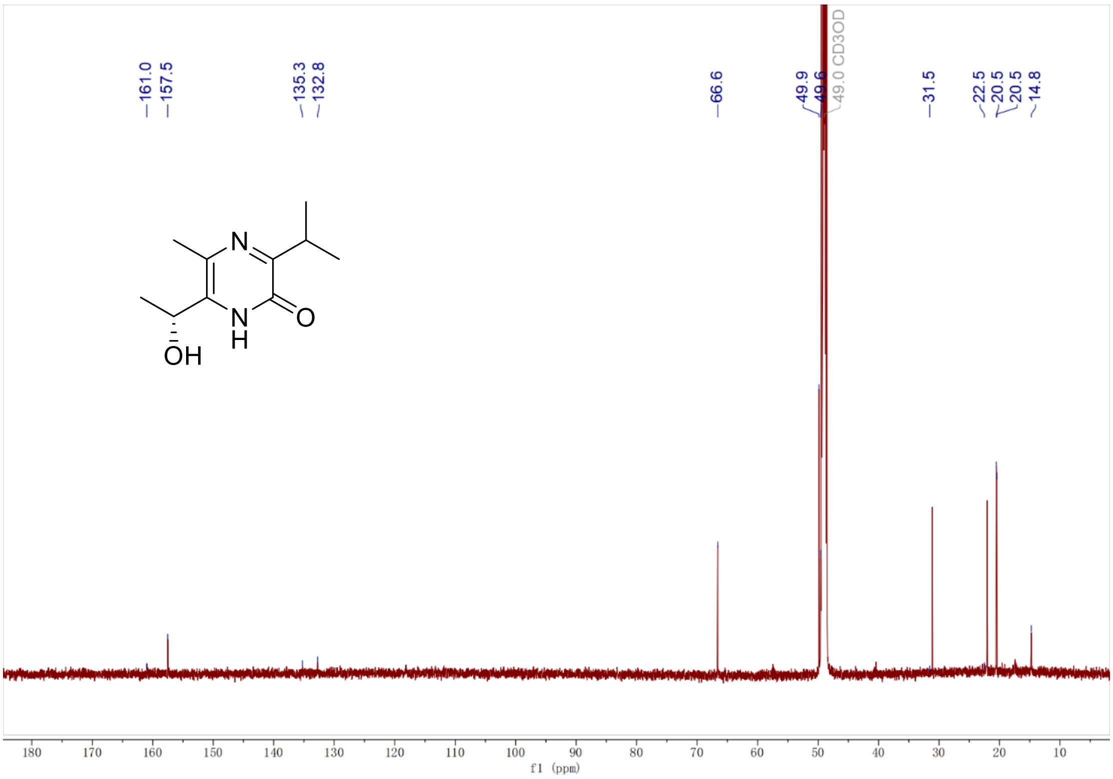

Figure S13. ^{13}C NMR spectrum of **2** in methanol-d_4.

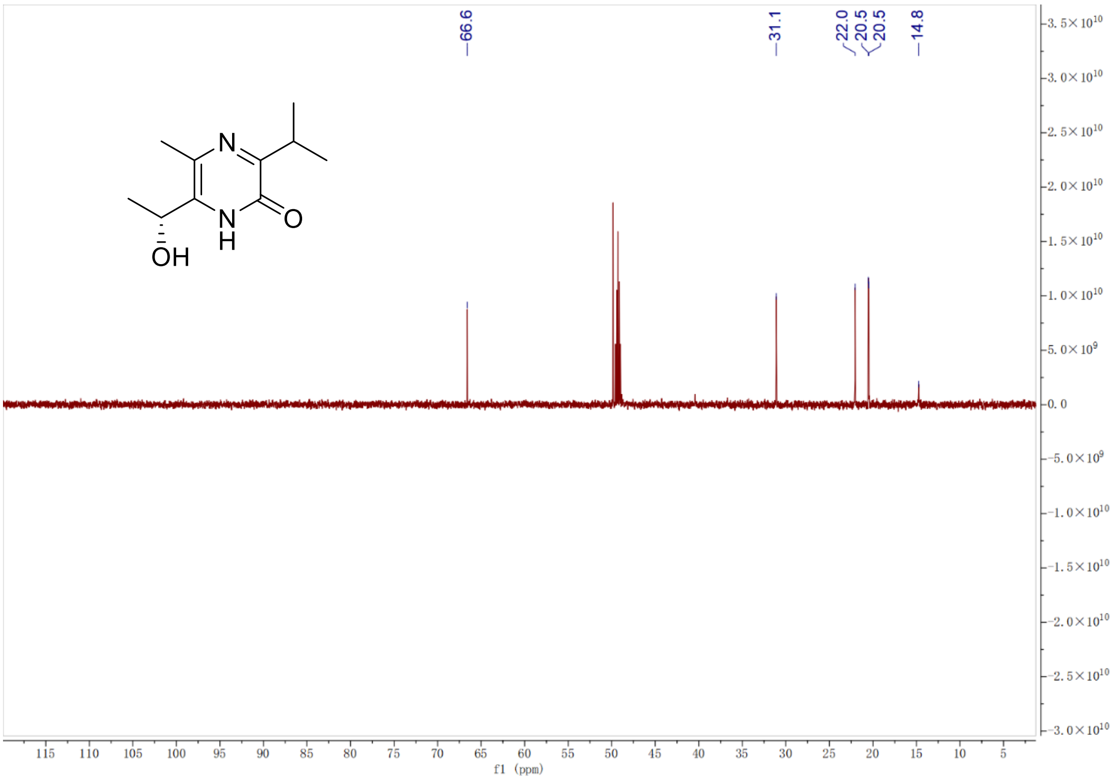

Figure S14. DEPT-135° spectrum of **2** in methanol-d_4.

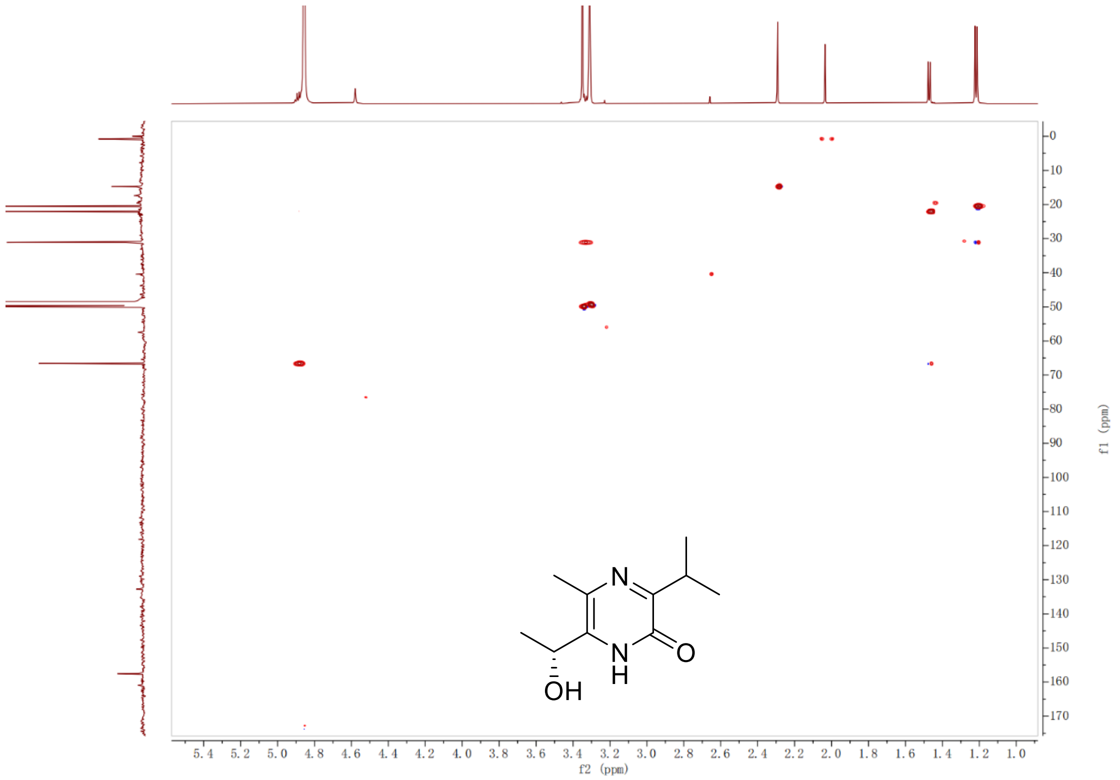

Figure S15. HSQC spectrum of **2** in methanol-d_4.

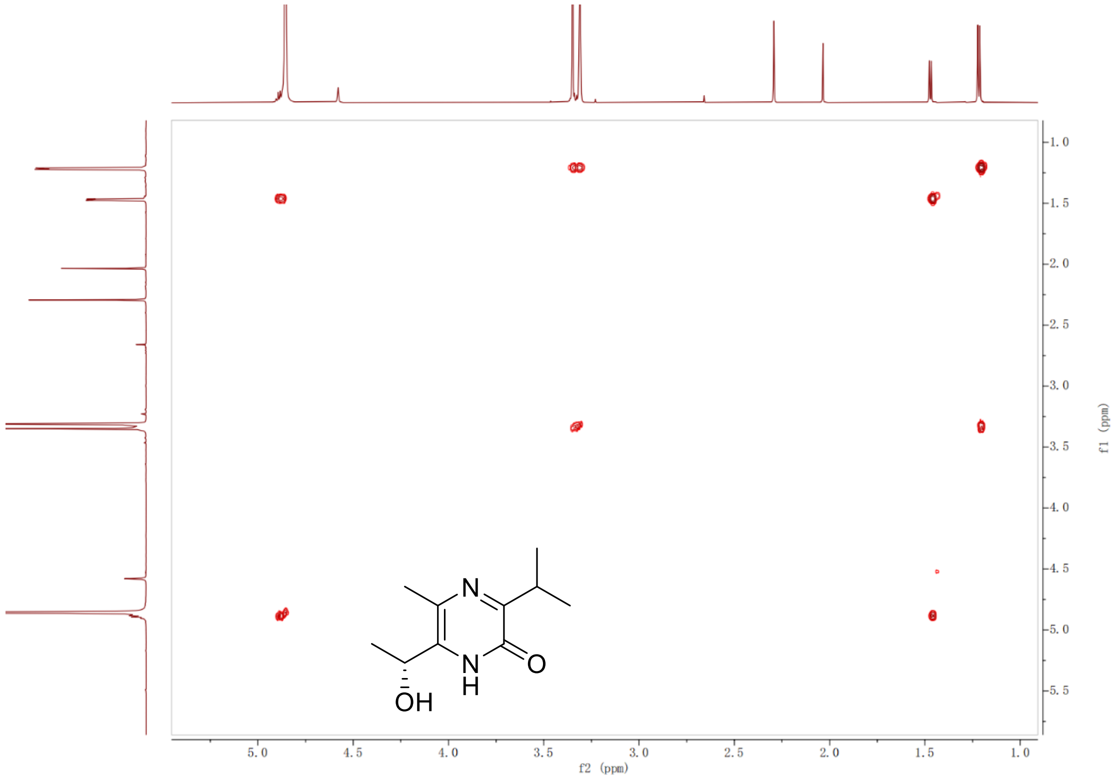

Figure S16. ^{1}H–^{1}H COSY spectrum of **2** in methanol-d_4.

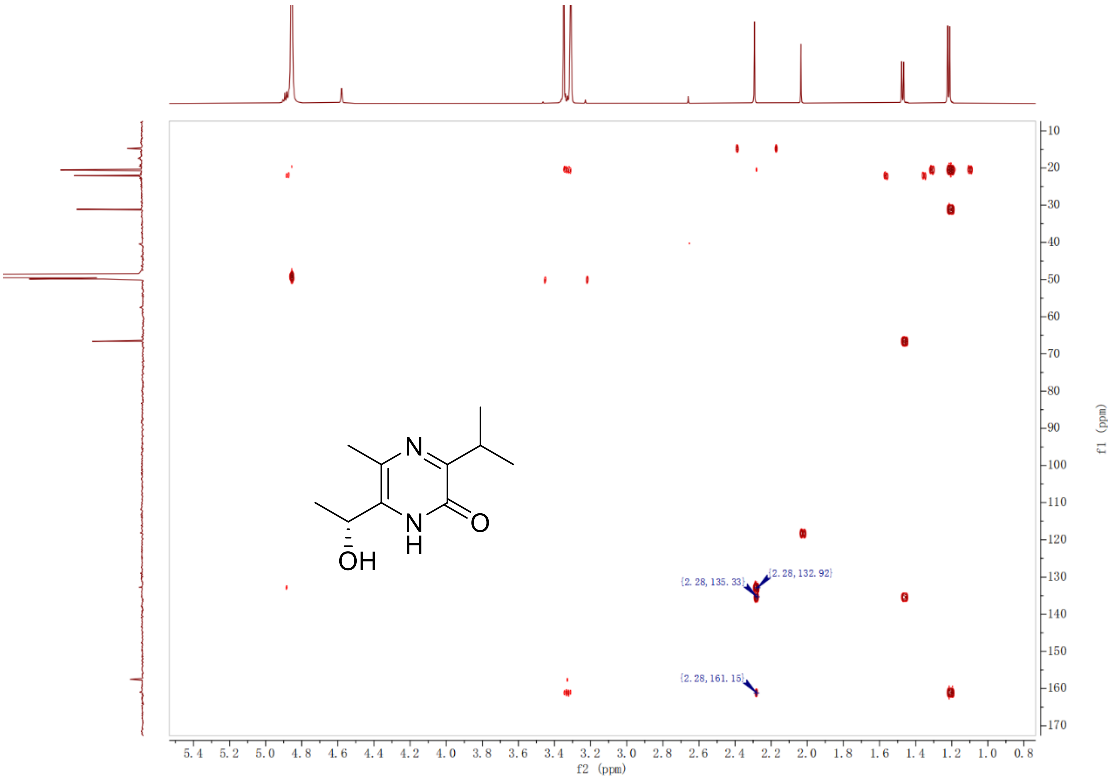

Figure S17. HMBC spectrum of **2** in methanol-d_4.

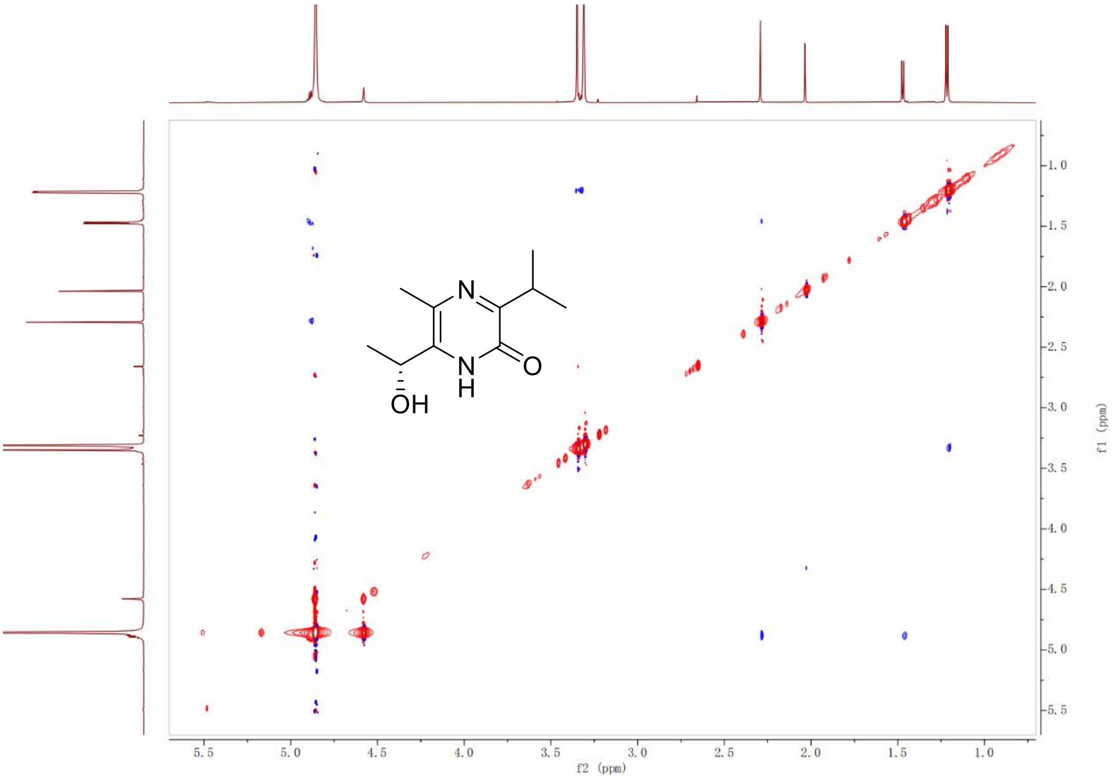

Figure S18. NOESY spectrum of **2** in methanol-d_4.

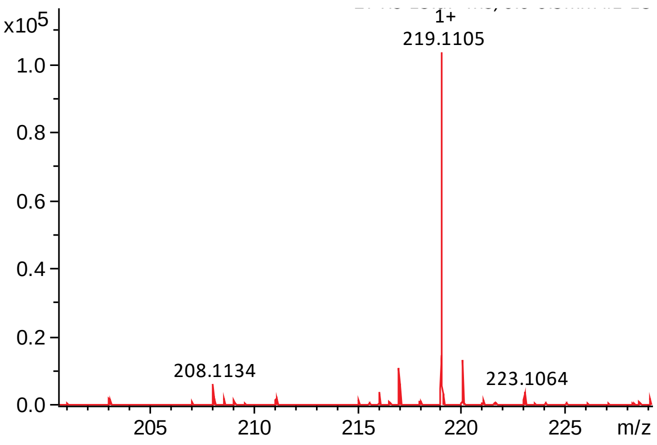

Figure S19. (+)-HRESIMS spectrum of **2**.

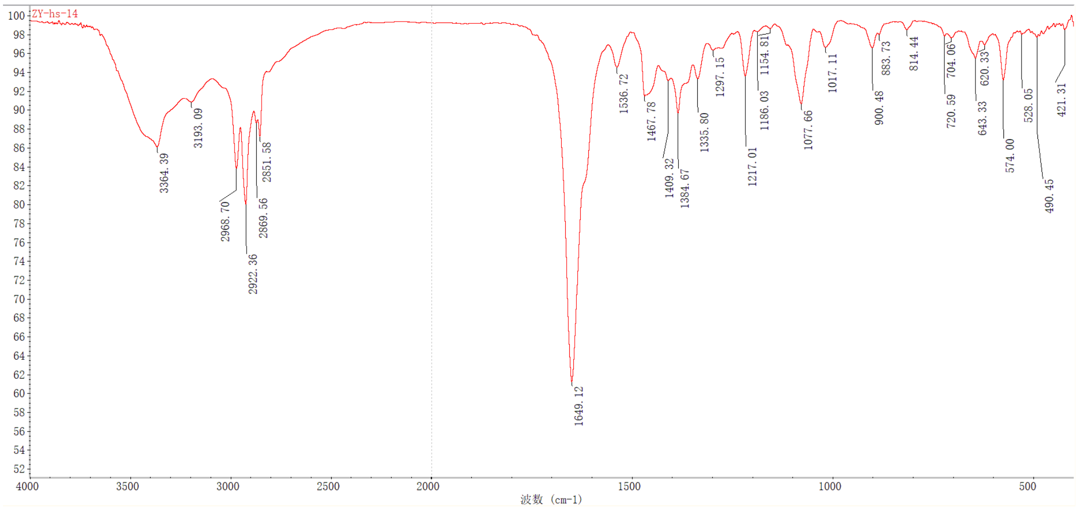

Figure S20. IR spectrum of **2**.

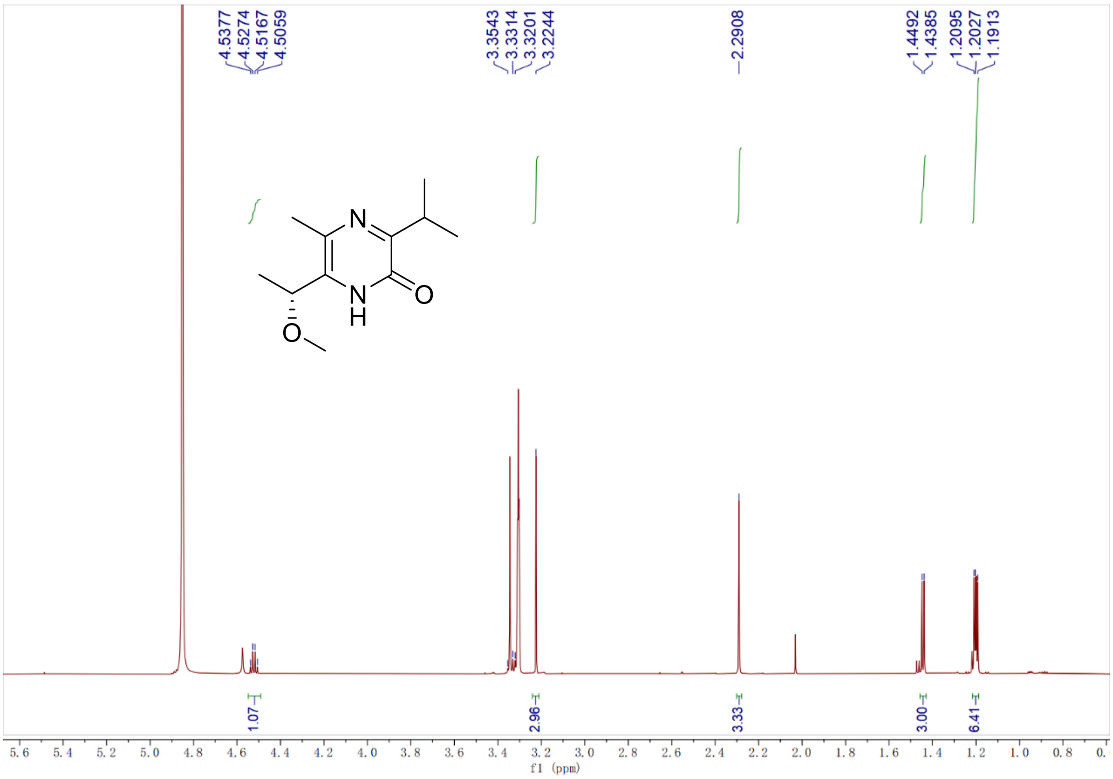

Figure S21. ^{1}H NMR spectrum of **3** in methanol-d_4.

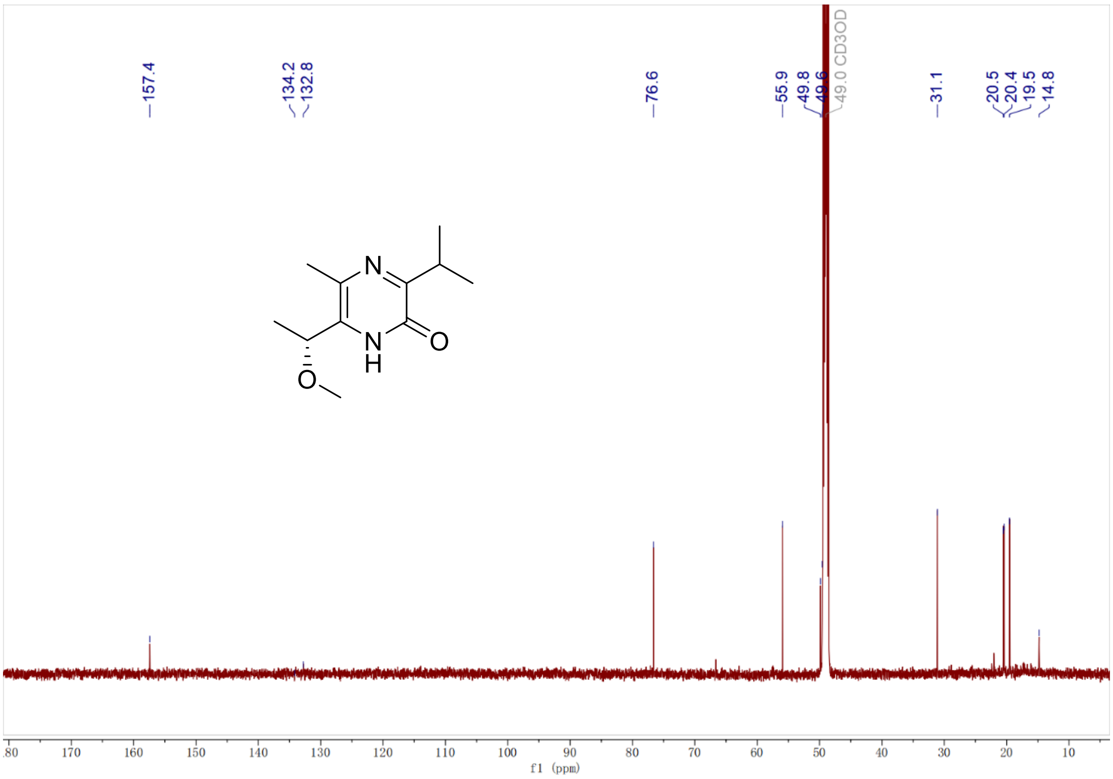

Figure S22. ^{13}C NMR spectrum of **3** in methanol-d_4.

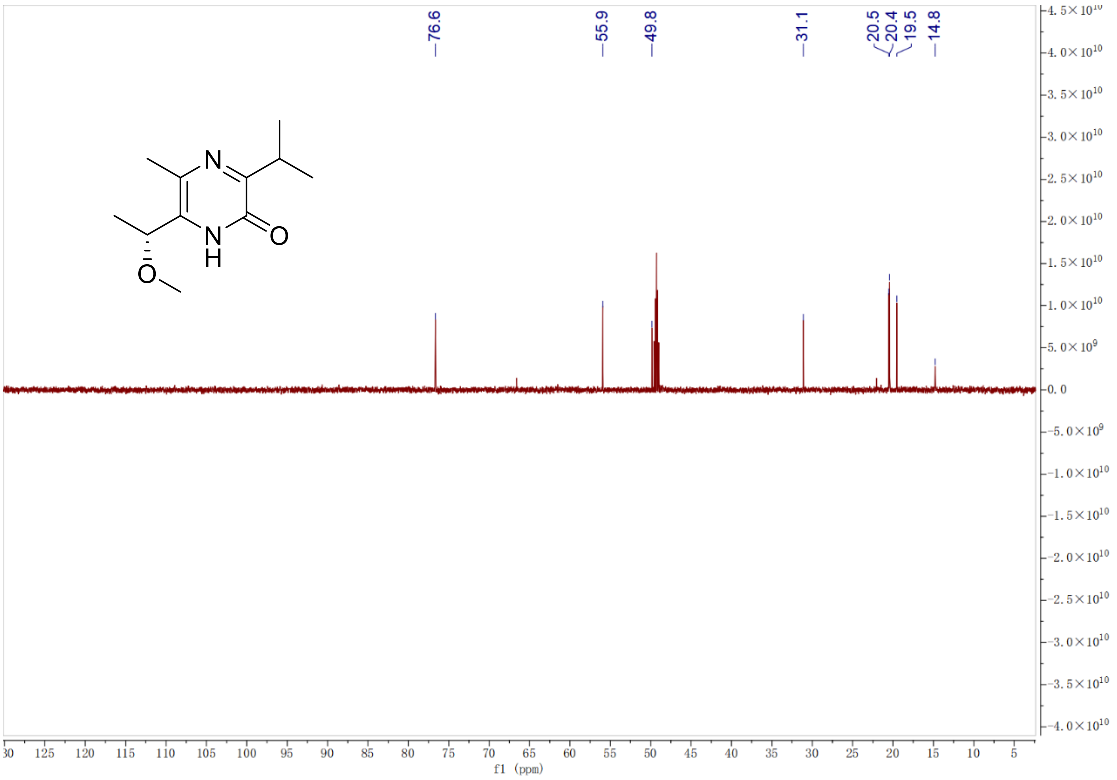

Figure S23. DEPT-135° spectrum of **3** in methanol-d_4.

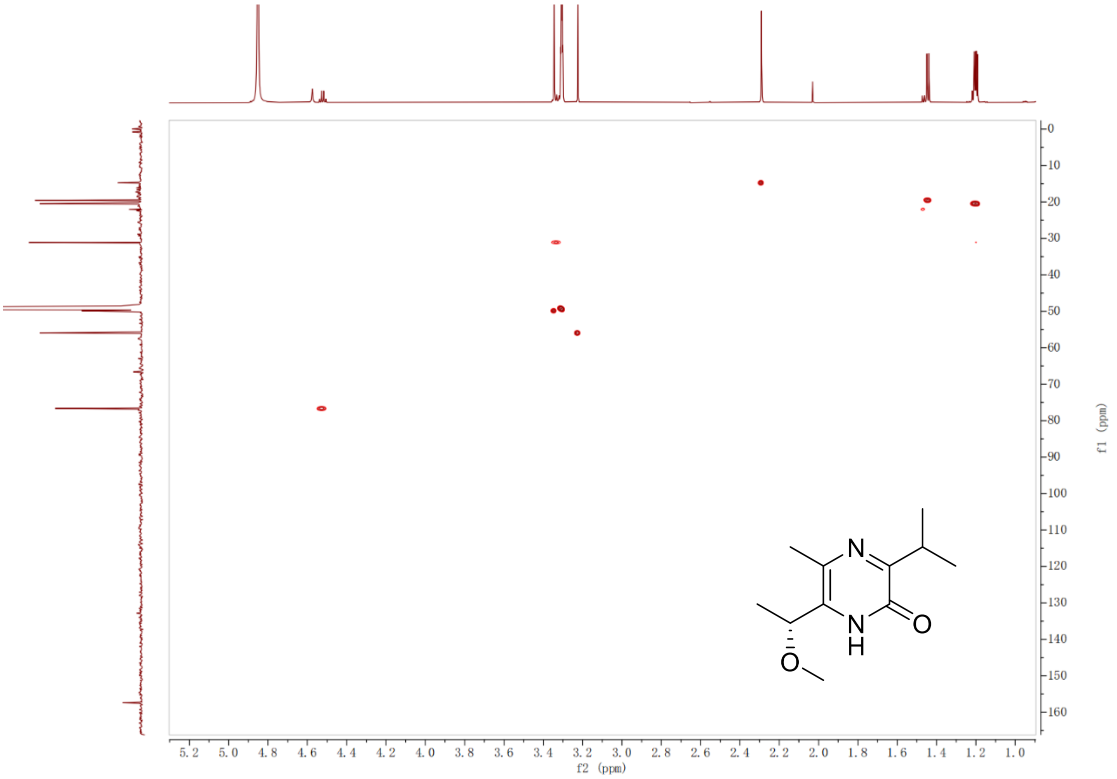

Figure S24. HSQC spectrum of **3** in methanol-d_4.

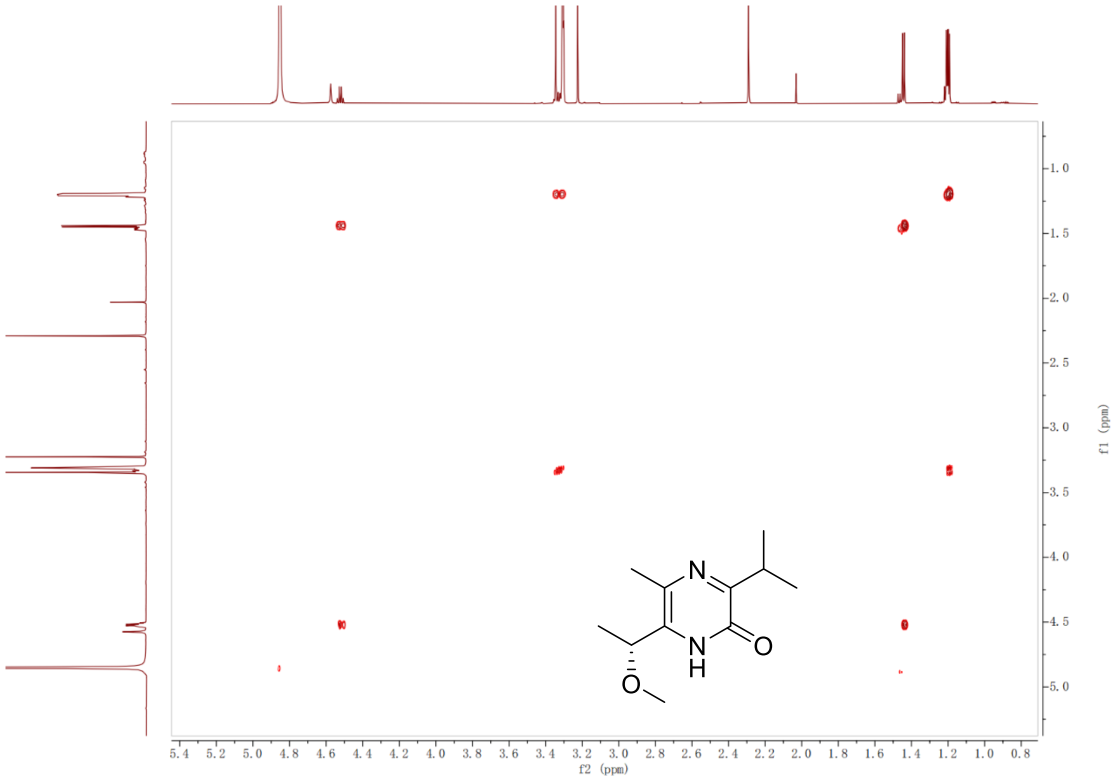

Figure S25. ^{1}H–^{1}H COSY spectrum of **3** in methanol-d_4.

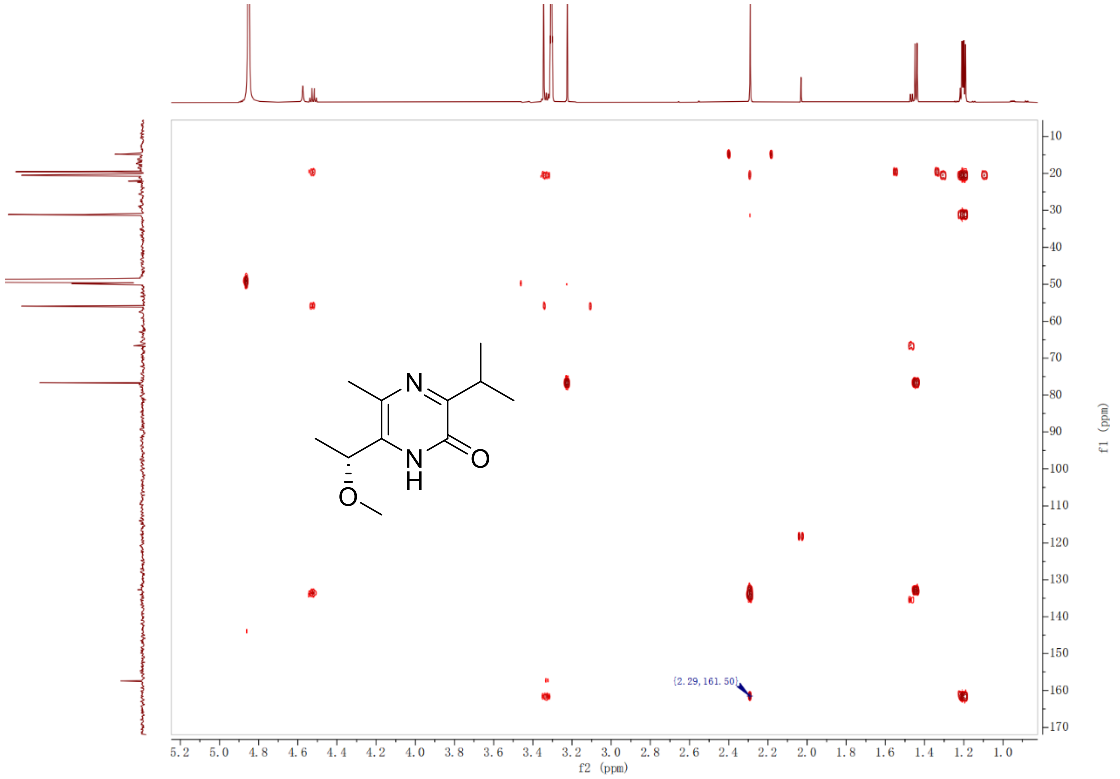

Figure S26. HMBC spectrum of **3** in methanol-d_4.

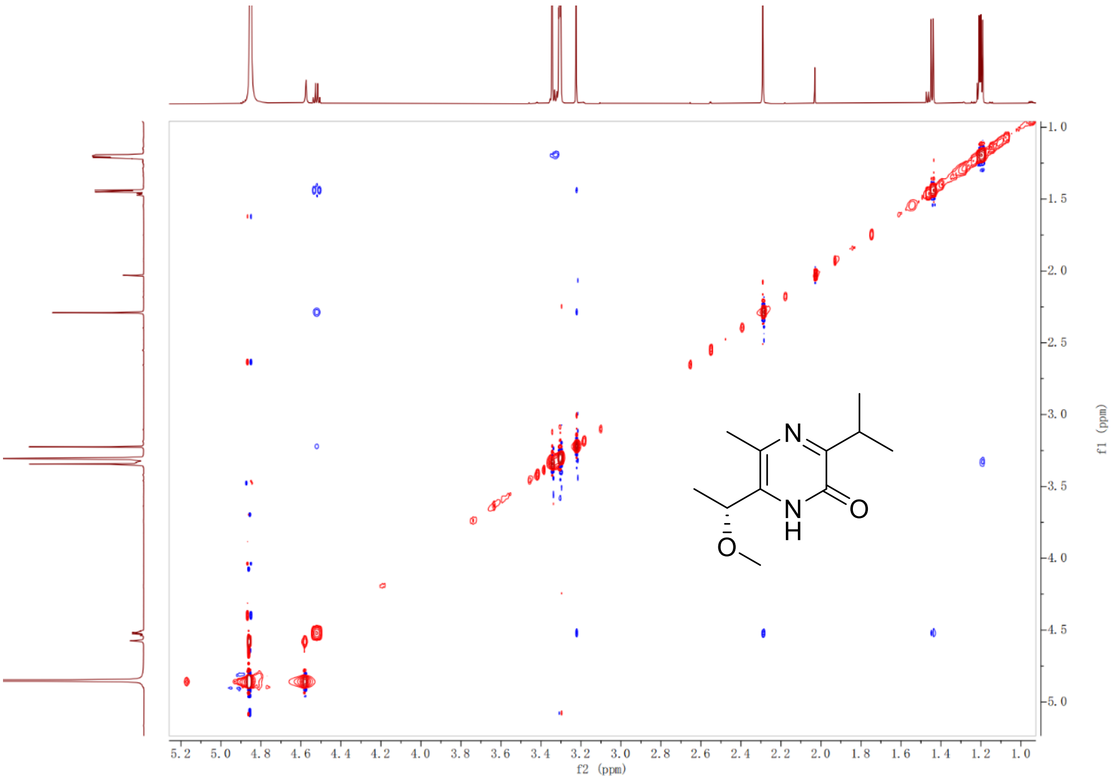

Figure S27. NOESY spectrum of **3** in methanol-d_4.

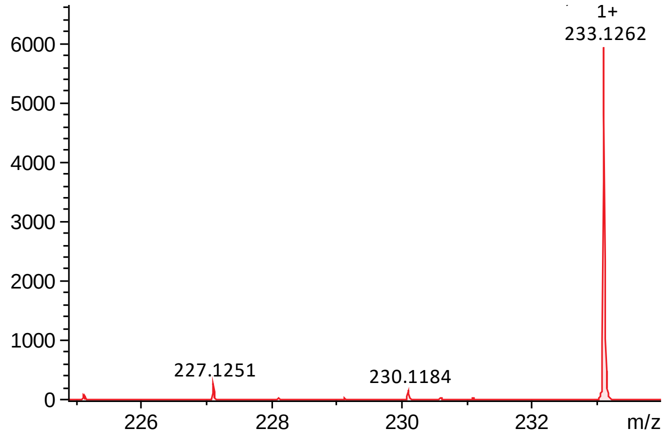

Figure S28. (+)-HR-ESI-MS spectrum of **3**.

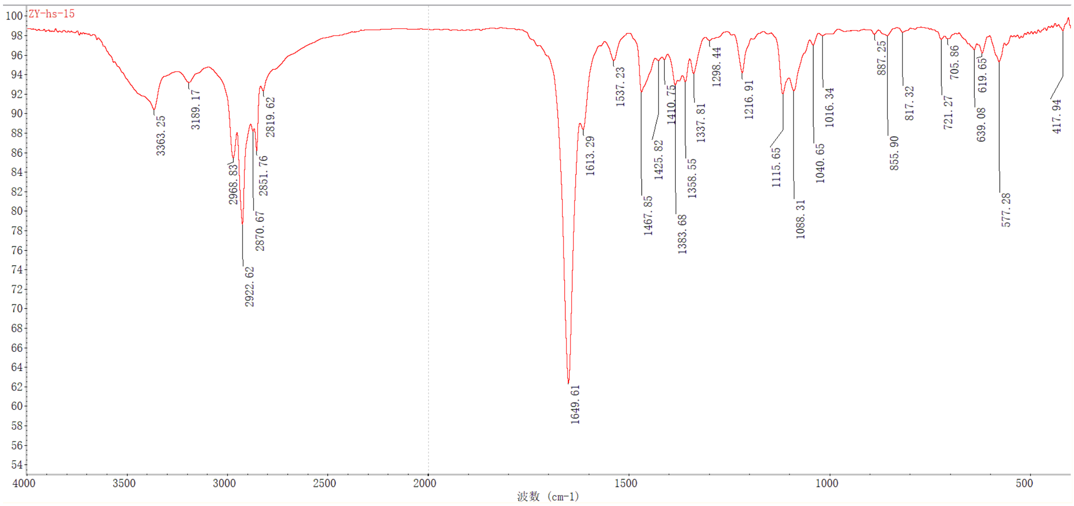

Figure S29. IR spectrum of **3**.

Details of theoretical computation for compound 2:

Table S1. Gibbs free energies[a] and equilibrium populations[b] of low-energy conformers of **2**.

Conformers	ΔG(a.u.)	P(%)/100	G(a.u.)
**2**A000001_tddft_	0.0	44.77	-650.469226
**2**A000002_tddft_	0.00056	24.88	-650.468671
**2**A000003_tddft_	0.00046	27.62	-650.46877
**2**A000005_tddft_	0.00613	0.07	-650.463099
**2**A000006_tddft_	0.00744	0.02	-650.461787
**2**A000008_tddft_	0.00477	0.29	-650.464453
**2**A000010_tddft_	0.00446	0.4	-650.464767
**2**A000011_tddft_	0.00437	0.44	-650.464855
**2**A000012_tddft_	0.00426	0.49	-650.464968
**2**A000013_tddft_	0.00461	0.34	-650.464616
**2**A000014_tddft_	0.00686	0.03	-650.462369
**2**A000016_tddft_	0.00646	0.05	-650.462768
**2**A000017_tddft_	0.00532	0.16	-650.463903
**2**A000018_tddft_	0.00476	0.29	-650.464469
**2**A000019_tddft_	0.00534	0.16	-650.463888

[a] wB97M-V/def2-TZVP, in a.u.

[b] From ΔG values at 298.15K.

Table S2. Cartesian coordinates for the low-energy reoptimized random research conformers of **2** at B3LYP-D3(BJ)/6-31G* level of theory in acetonitrile.

**2**A000001_en_		Standard Orientation (A.U.)			
Center number	Atomic number	Atomic Type	X	Y	Z
0	6	0	-13.758314	0.843825	-2.001674
1	6	0	-12.60436	0.056902	0.181202
2	7	0	-13.893548	-1.560645	1.728043

3	6	0	-16.29929	-2.477843	1.286652
4	6	0	-17.37562	-1.528858	-1.091279
5	7	0	-16.14647	0.00723	-2.584121
6	6	0	-10.037067	0.895876	1.16108
7	6	0	-12.580413	2.665861	-3.840209
8	8	0	-17.345651	-3.948777	2.773691
9	6	0	-20.024685	-2.376229	-1.741823
10	6	0	-21.946457	-0.907278	-0.113726
11	6	0	-10.222689	3.447592	2.498033
12	6	0	-20.620731	-2.117678	-4.557991
13	8	0	-9.092266	-0.877132	2.967962
14	1	0	-12.971466	-2.166422	3.303415
15	1	0	-8.727519	1.048424	-0.436122
16	1	0	-13.214917	4.609377	-3.488009
17	1	0	-10.51601	2.660598	-3.744575
18	1	0	-13.147163	2.170366	-5.765214
19	1	0	-20.158463	-4.371955	-1.19364
20	1	0	-21.540478	-1.140928	1.899345
21	1	0	-21.883966	1.112937	-0.568783
22	1	0	-23.866718	-1.592361	-0.47513
23	1	0	-11.517437	3.313397	4.106141
24	1	0	-8.356919	4.012431	3.185192
25	1	0	-10.92818	4.89618	1.204194
26	1	0	-22.522501	-2.836641	-4.947159
27	1	0	-19.271917	-3.178638	-5.714607
28	1	0	-20.537296	-0.138906	-5.152609
29	1	0	-8.477517	-2.346715	2.055313

**2**A000002_en_		Standard Orientation (A.U.)			
Center number	Atomic number	Atomic Type	X	Y	Z
0	6	0	-12.90799	-1.903924	0.229734
1	6	0	-12.86278	0.68002	0.431715
2	7	0	-15.025834	1.985106	-0.104007
3	6	0	-17.292353	0.912492	-0.836291
4	6	0	-17.149473	-1.853971	-1.015996
5	7	0	-15.085997	-3.110712	-0.502862
6	6	0	-10.57894	2.281825	1.070515
7	6	0	-10.649559	-3.565688	0.702963
8	8	0	-19.185084	2.218113	-1.266519
9	6	0	-19.541034	-3.2305	-1.754769
10	6	0	-19.030381	-5.890397	-2.763069
11	6	0	-8.868084	2.612571	-1.241518
12	6	0	-21.376782	-3.291277	0.509255
13	8	0	-11.520948	4.652969	1.956785
14	1	0	-14.965714	3.891902	0.130888
15	1	0	-9.513369	1.343548	2.583248
16	1	0	-9.267986	-2.681005	1.959482
17	1	0	-11.266043	-5.34413	1.557397
18	1	0	-9.673004	-4.034558	-1.066331
19	1	0	-20.439903	-2.096045	-3.240389
20	1	0	-17.749381	-5.847901	-4.387961
21	1	0	-18.160999	-7.076781	-1.309393
22	1	0	-20.805758	-6.776359	-3.353317
23	1	0	-9.914408	3.519991	-2.778226

24	1	0	-7.231823	3.786435	-0.756357
25	1	0	-8.16788	0.784605	-1.905522
26	1	0	-21.772461	-1.380025	1.188861
27	1	0	-20.566802	-4.391727	2.066733
28	1	0	-23.168059	-4.172056	-0.041459
29	1	0	-10.156885	5.872921	1.859092

**2**A000003_en_		Standard Orientation (A.U.)			
Center number	Atomic number	Atomic Type	X	Y	Z
0	6	0	-13.35888	-1.077306	1.500968
1	6	0	-13.200304	1.292643	0.462592
2	7	0	-15.192429	2.148192	-0.940844
3	6	0	-17.381009	0.807147	-1.426804
4	6	0	-17.371581	-1.704866	-0.245235
5	7	0	-15.477904	-2.524653	1.112038
6	6	0	-10.944045	3.045418	0.601354
7	6	0	-11.284781	-2.269359	3.03908
8	8	0	-19.115062	1.711663	-2.709858
9	6	0	-19.656453	-3.358072	-0.697625
10	6	0	-19.539751	-4.467122	-3.391665
11	6	0	-8.92989	2.291981	-1.337816
12	6	0	-19.937637	-5.442729	1.284335
13	8	0	-11.875653	5.539418	0.147225
14	1	0	-15.064094	3.940032	-1.624361
15	1	0	-10.134987	2.935782	2.508405
16	1	0	-10.104356	-0.866761	3.993759
17	1	0	-12.11545	-3.508398	4.469284
18	1	0	-10.03735	-3.433141	1.858534
19	1	0	-21.31242	-2.111831	-0.620773
20	1	0	-21.247185	-5.568511	-3.790428
21	1	0	-19.390355	-2.964604	-4.80313
22	1	0	-17.901427	-5.720207	-3.586764
23	1	0	-7.314633	3.584079	-1.230531
24	1	0	-8.24081	0.377089	-0.975556
25	1	0	-9.719965	2.362734	-3.248205
26	1	0	-21.663454	-6.528928	0.928192
27	1	0	-20.043452	-4.656325	3.195627
28	1	0	-18.327734	-6.739306	1.227205
29	1	0	-10.445985	6.605895	-0.2736

**2**A000005_en_		Standard Orientation (A.U.)			
Center number	Atomic number	Atomic Type	X	Y	Z
0	6	0	-13.872888	0.798349	-2.446902
1	6	0	-13.083831	1.248567	-0.014827
2	7	0	-14.297422	-0.032392	1.886746
3	6	0	-16.244513	-1.75623	1.582491
4	6	0	-16.950545	-2.092722	-1.080302
5	7	0	-15.810662	-0.87089	-2.901979
6	6	0	-10.936297	2.962824	0.834754
7	6	0	-12.722574	1.9909	-4.759033
8	8	0	-17.205989	-2.815724	3.432803
9	6	0	-19.061573	-3.896402	-1.762873
10	6	0	-18.41966	-6.63306	-1.014566

11	6	0	-8.354634	1.814199	0.253257
12	6	0	-21.604521	-3.053801	-0.629249
13	8	0	-11.012364	5.389709	-0.326497
14	1	0	-13.767078	0.270193	3.707012
15	1	0	-11.107812	3.182167	2.897245
16	1	0	-14.225206	2.634651	-6.028347
17	1	0	-11.526498	3.594726	-4.274715
18	1	0	-11.592413	0.604091	-5.808094
19	1	0	-19.204274	-3.801052	-3.82654
20	1	0	-18.23138	-6.802065	1.035154
21	1	0	-19.92285	-7.909861	-1.645974
22	1	0	-16.645934	-7.246469	-1.889543
23	1	0	-6.866656	3.079505	0.931983
24	1	0	-8.153785	-0.014507	1.196299
25	1	0	-8.118689	1.539269	-1.781284
26	1	0	-22.084664	-1.13047	-1.227763
27	1	0	-21.532452	-3.098669	1.434308
28	1	0	-23.115553	-4.320107	-1.263029
29	1	0	-12.668333	6.115484	-0.00976

**2**A000006_en_		Standard Orientation (A.U.)			
Center number	Atomic number	Atomic Type	X	Y	Z
0	6	0	-12.809766	-0.907566	0.839088
1	6	0	-13.278289	1.468674	-0.09744
2	7	0	-15.605814	1.898714	-1.144541
3	6	0	-17.550308	0.150158	-1.346609
4	6	0	-16.855416	-2.323212	-0.30812
5	7	0	-14.631097	-2.747554	0.690867
6	6	0	-11.510214	3.736601	-0.03366
7	6	0	-10.348305	-1.691432	2.043561
8	8	0	-19.589905	0.743033	-2.325603
9	6	0	-18.744609	-4.468683	-0.379321
10	6	0	-21.118561	-3.862961	1.188698
11	6	0	-11.661736	5.114349	2.506298
12	6	0	-19.435077	-5.204054	-3.10763
13	8	0	-8.987031	3.087667	-0.701514
14	1	0	-16.00781	3.632235	-1.866477
15	1	0	-12.130812	5.034546	-1.524697
16	1	0	-10.604701	-3.548314	2.906958
17	1	0	-8.82038	-1.830895	0.653993
18	1	0	-9.737547	-0.385517	3.530422
19	1	0	-17.772661	-6.071006	0.501705
20	1	0	-22.129041	-2.251216	0.38452
21	1	0	-22.391444	-5.496359	1.20642
22	1	0	-20.620973	-3.414503	3.148091
23	1	0	-13.598925	5.728529	2.889457
24	1	0	-11.067916	3.877255	4.056805
25	1	0	-10.432061	6.776337	2.470991
26	1	0	-20.701719	-6.842364	-3.100502
27	1	0	-17.744296	-5.703235	-4.193809
28	1	0	-20.389083	-3.641011	-4.062906
29	1	0	-8.110102	2.558226	0.818076

**2**A000008_en_		Standard Orientation (A.U.)			
Center number	Atomic number	Atomic Type	X	Y	Z
0	6	0	-12.835003	-0.940583	0.050803
1	6	0	-13.364173	1.585017	-0.223434
2	7	0	-15.835414	2.252752	-0.655816
3	6	0	-17.851776	0.59748	-0.835079
4	6	0	-17.083309	-2.049508	-0.524888
5	7	0	-14.739539	-2.701309	-0.117214
6	6	0	-11.524784	3.770663	0.001183
7	6	0	-10.247656	-1.994886	0.584749
8	8	0	-20.027189	1.36072	-1.223777
9	6	0	-19.127097	-4.029884	-0.764198
10	6	0	-19.836102	-4.36872	-3.572382
11	6	0	-10.666357	4.17226	2.735998
12	6	0	-18.386793	-6.549196	0.443054
13	8	0	-9.455248	3.303642	-1.664331
14	1	0	-16.27986	4.105897	-0.883377
15	1	0	-12.525097	5.486311	-0.619953
16	1	0	-8.76949	-0.609074	0.22246
17	1	0	-10.108109	-2.62996	2.55429
18	1	0	-9.916487	-3.650548	-0.61192
19	1	0	-20.799031	-3.271598	0.201297
20	1	0	-21.401326	-5.711094	-3.759202
21	1	0	-20.416803	-2.571139	-4.410626
22	1	0	-18.221354	-5.105055	-4.641201
23	1	0	-9.664277	2.511943	3.448025
24	1	0	-9.403266	5.810229	2.849726
25	1	0	-12.295879	4.537089	3.957117
26	1	0	-17.907432	-6.315204	2.442428
27	1	0	-16.742424	-7.369501	-0.505443
28	1	0	-19.958269	-7.888853	0.300291
29	1	0	-8.189385	4.588399	-1.32456

**2**A000010_en_		Standard Orientation (A.U.)			
Center number	Atomic number	Atomic Type	X	Y	Z
0	6	0	-14.025867	0.480404	-2.405294
1	6	0	-13.24242	1.420341	-0.117707
2	7	0	-14.400087	0.519049	2.014374
3	6	0	-16.318503	-1.262054	2.054261
4	6	0	-17.033096	-2.119789	-0.484114
5	7	0	-15.929303	-1.275841	-2.527048
6	6	0	-11.146113	3.351889	0.149196
7	6	0	-12.885666	1.314127	-4.877226
8	8	0	-17.26718	-1.981761	4.066736
9	6	0	-19.085586	-4.09782	-0.654575
10	6	0	-20.341921	-4.180951	-3.256272
11	6	0	-11.126411	4.77835	2.658584
12	6	0	-17.995391	-6.700899	0.079471
13	8	0	-8.823513	2.000424	-0.227181
14	1	0	-13.862553	1.169869	3.735607
15	1	0	-11.405724	4.725317	-1.383986
16	1	0	-13.324202	-0.086697	-6.329307

17	1	0	-13.659029	3.134903	-5.504098
18	1	0	-10.834656	1.510802	-4.733021
19	1	0	-20.503245	-3.602349	0.775727
20	1	0	-18.978081	-4.705851	-4.719098
21	1	0	-21.870895	-5.576257	-3.257867
22	1	0	-21.136723	-2.340273	-3.768874
23	1	0	-9.649299	6.224919	2.609905
24	1	0	-12.932118	5.727673	3.007575
25	1	0	-10.697396	3.513921	4.241286
26	1	0	-17.146385	-6.642512	1.962913
27	1	0	-16.545677	-7.285743	-1.279953
28	1	0	-19.493058	-8.130522	0.083416
29	1	0	-7.493259	3.255998	-0.386714

**2**A000011_en_		Standard Orientation (A.U.)			
Center number	Atomic number	Atomic Type	X	Y	Z
0	6	0	-13.00286	-0.777176	-1.706351
1	6	0	-13.087646	1.369165	-0.246783
2	7	0	-15.305206	1.89525	0.983362
3	6	0	-17.480522	0.440295	0.874622
4	6	0	-17.1939	-1.788407	-0.747564
5	7	0	-15.083928	-2.305683	-1.926041
6	6	0	-10.837294	3.120705	0.114718
7	6	0	-10.67337	-1.559563	-3.143907
8	8	0	-19.408538	1.050334	2.049077
9	6	0	-19.446066	-3.529831	-0.970943
10	6	0	-19.339076	-5.195449	-3.330707
11	6	0	-11.485381	5.72139	1.185334
12	6	0	-19.682503	-5.136998	1.450622
13	8	0	-8.915286	1.905666	1.587404
14	1	0	-15.443976	3.472767	2.064055
15	1	0	-9.963522	3.37346	-1.74088
16	1	0	-8.97546	-1.380606	-1.981474
17	1	0	-10.87914	-3.524581	-3.742247
18	1	0	-10.397876	-0.404702	-4.845991
19	1	0	-21.124364	-2.315022	-1.065465
20	1	0	-19.193587	-4.055927	-5.051975
21	1	0	-17.70138	-6.456438	-3.271896
22	1	0	-21.050425	-6.353129	-3.45605
23	1	0	-9.765933	6.863179	1.24231
24	1	0	-12.88156	6.701927	0.013651
25	1	0	-12.208887	5.598081	3.124492
26	1	0	-19.811225	-3.934278	3.126482
27	1	0	-18.038222	-6.380479	1.655453
28	1	0	-21.380409	-6.318751	1.36442
29	1	0	-9.625794	1.556218	3.24458

**2**A000012_en_		Standard Orientation (A.U.)			
Center number	Atomic number	Atomic Type	X	Y	Z
0	6	0	-12.695025	-1.129563	-0.742345
1	6	0	-13.122306	1.385742	-0.271779
2	7	0	-15.557683	2.125154	0.198563
3	6	0	-17.643041	0.542448	0.20448

4	6	0	-16.983186	-2.097494	-0.328716
5	7	0	-14.660403	-2.821004	-0.759239
6	6	0	-11.034948	3.344776	-0.201808
7	6	0	-10.099277	-2.17234	-1.270378
8	8	0	-19.790609	1.363073	0.631461
9	6	0	-19.132033	-3.976048	-0.418678
10	6	0	-20.613376	-3.648436	-2.908952
11	6	0	-11.925071	6.077728	-0.466335
12	6	0	-18.244153	-6.704794	-0.075642
13	8	0	-9.725219	2.968161	2.144569
14	1	0	-15.939793	3.968799	0.558922
15	1	0	-9.762737	2.913478	-1.783076
16	1	0	-9.541571	-1.87606	-3.247173
17	1	0	-8.672581	-1.288803	-0.066849
18	1	0	-10.105762	-4.207243	-0.919868
19	1	0	-20.419052	-3.474243	1.128735
20	1	0	-22.252166	-4.913409	-2.939851
21	1	0	-21.28627	-1.705519	-3.117118
22	1	0	-19.410108	-4.108788	-4.531354
23	1	0	-13.062057	6.661073	1.163194
24	1	0	-10.277929	7.325769	-0.544315
25	1	0	-13.015219	6.360028	-2.202871
26	1	0	-17.210594	-6.943836	1.701046
27	1	0	-16.985647	-7.271668	-1.615379
28	1	0	-19.873796	-7.980954	-0.060925
29	1	0	-8.19887	3.986496	2.074093

**2**A000013_en_		Standard Orientation (A.U.)			
Center number	Atomic number	Atomic Type	X	Y	Z
0	6	0	-14.62495	2.06902	0.081818
1	6	0	-12.635042	0.400851	0.117161
2	7	0	-13.117392	-2.054476	-0.55152
3	6	0	-15.459901	-3.0057	-1.235866
4	6	0	-17.448473	-1.075555	-1.200333
5	7	0	-17.001454	1.274728	-0.580303
6	6	0	-9.94599	1.168301	0.780145
7	6	0	-14.331471	4.817789	0.76245
8	8	0	-15.737628	-5.257806	-1.800763
9	6	0	-20.093845	-1.927885	-1.856614
10	6	0	-21.230338	-3.402416	0.387766
11	6	0	-8.186566	-1.022094	1.441056
12	6	0	-21.806748	0.260253	-2.650549
13	8	0	-8.893112	2.718458	-1.176668
14	1	0	-11.697904	-3.343317	-0.537436
15	1	0	-10.062659	2.429778	2.412553
16	1	0	-12.636332	5.63018	-0.093317
17	1	0	-15.987215	5.856038	0.097075
18	1	0	-14.196583	5.090272	2.815305
19	1	0	-19.909928	-3.261809	-3.434413
20	1	0	-23.099922	-4.139086	-0.111076
21	1	0	-20.021705	-4.994584	0.912609
22	1	0	-21.441683	-2.162397	2.033893
23	1	0	-6.356648	-0.254059	2.011372
24	1	0	-8.934833	-2.154949	3.003313

25	1	0	-7.853509	-2.264835	-0.184864
26	1	0	-23.669011	-0.461849	-3.193837
27	1	0	-21.012825	1.295073	-4.257113
28	1	0	-22.055625	1.60433	-1.098959
29	1	0	-8.84622	1.714363	-2.713832

**2**A000014_en_		Standard Orientation (A.U.)			
Center number	Atomic number	Atomic Type	X	Y	Z
0	6	0	-14.352952	1.689837	0.966899
1	6	0	-12.638507	0.108506	-0.171534
2	7	0	-13.579546	-1.867913	-1.56133
3	6	0	-16.112527	-2.411005	-1.943141
4	6	0	-17.784557	-0.617275	-0.646039
5	7	0	-16.900435	1.262169	0.69492
6	6	0	-9.783666	0.355191	-0.16118
7	6	0	-13.657852	3.971726	2.51642
8	8	0	-16.7597	-4.243818	-3.244784
9	6	0	-20.619448	-0.933134	-0.845411
10	6	0	-21.539512	-0.655246	-3.590933
11	6	0	-8.898327	2.598436	-1.761315
12	6	0	-21.506966	-3.444636	0.323374
13	8	0	-8.963353	0.539048	2.401298
14	1	0	-12.359434	-3.08186	-2.410686
15	1	0	-9.009124	-1.383557	-1.002275
16	1	0	-14.025993	5.71668	1.457391
17	1	0	-14.833241	4.034452	4.218392
18	1	0	-11.677765	3.93647	3.077774
19	1	0	-21.413638	0.621147	0.268891
20	1	0	-20.967061	1.169183	-4.385765
21	1	0	-20.761512	-2.158149	-4.774739
22	1	0	-23.6063	-0.765738	-3.659099
23	1	0	-9.626641	4.378416	-1.006971
24	1	0	-6.828658	2.680924	-1.778469
25	1	0	-9.545092	2.392127	-3.715483
26	1	0	-23.573895	-3.559479	0.267549
27	1	0	-20.907939	-3.597681	2.299629
28	1	0	-20.731554	-5.048322	-0.721787
29	1	0	-7.174538	0.948516	2.366247

**2**A000016_en_		Standard Orientation (A.U.)			
Center number	Atomic number	Atomic Type	X	Y	Z
0	6	0	-14.391432	2.363819	-0.845819
1	6	0	-12.813335	0.67927	0.344697
2	7	0	-13.771264	-1.656851	0.908176
3	6	0	-16.211652	-2.472074	0.399314
4	6	0	-17.743553	-0.527799	-0.848294
5	7	0	-16.834056	1.708095	-1.404643
6	6	0	-10.073966	1.26253	0.992579
7	6	0	-13.5624	4.9869	-1.577438
8	8	0	-16.892054	-4.630626	0.992624
9	6	0	-20.465222	-1.091848	-1.518903
10	6	0	-20.672353	-3.264145	-3.442361
11	6	0	-8.873445	-0.539473	2.900682

12	6	0	-22.067744	-1.61678	0.851507
13	8	0	-8.582305	1.444199	-1.260865
14	1	0	-12.669493	-2.949233	1.798337
15	1	0	-10.032318	3.173434	1.778308
16	1	0	-13.542363	6.26781	0.055316
17	1	0	-11.665843	4.97622	-2.394453
18	1	0	-14.889473	5.75305	-2.961014
19	1	0	-21.170171	0.638652	-2.411634
20	1	0	-22.651096	-3.540383	-3.987146
21	1	0	-19.586448	-2.846519	-5.155343
22	1	0	-19.962373	-5.025173	-2.629928
23	1	0	-6.962172	0.117858	3.32286
24	1	0	-9.947586	-0.60236	4.668892
25	1	0	-8.71255	-2.464826	2.148207
26	1	0	-24.049675	-1.893797	0.318889
27	1	0	-21.974518	-0.029669	2.178257
28	1	0	-21.403068	-3.31721	1.817134
29	1	0	-8.630869	-0.192739	-2.092167

**2**A000017_en_		Standard Orientation (A.U.)			
Center number	Atomic number	Atomic Type	X	Y	Z
0	6	0	-13.588182	-0.810511	1.161787
1	6	0	-13.034206	1.164049	-0.430412
2	7	0	-14.797873	1.788849	-2.222779
3	6	0	-17.100228	0.592287	-2.580351
4	6	0	-17.4963	-1.494851	-0.801854
5	7	0	-15.827586	-2.098273	0.915453
6	6	0	-10.679638	2.816191	-0.385481
7	6	0	-11.852071	-1.728636	3.227628
8	8	0	-18.584992	1.283572	-4.248104
9	6	0	-19.917009	-2.987383	-1.067395
10	6	0	-19.731281	-4.751629	-3.381474
11	6	0	-10.919193	4.939438	1.565663
12	6	0	-20.586669	-4.46225	1.326291
13	8	0	-8.426971	1.381165	-0.068202
14	1	0	-14.437077	3.234213	-3.43409
15	1	0	-10.498028	3.667444	-2.265882
16	1	0	-11.183828	-0.195432	4.449235
17	1	0	-12.892071	-3.062993	4.409757
18	1	0	-10.192399	-2.702811	2.464462
19	1	0	-21.41767	-1.611963	-1.466344
20	1	0	-19.306113	-3.679008	-5.096204
21	1	0	-18.239824	-6.161242	-3.09749
22	1	0	-21.521293	-5.75268	-3.665761
23	1	0	-12.581712	6.106314	1.176424
24	1	0	-11.115012	4.169167	3.477631
25	1	0	-9.235689	6.138259	1.499963
26	1	0	-20.744758	-3.208462	2.96495
27	1	0	-19.138927	-5.875676	1.754199
28	1	0	-22.395156	-5.436685	1.0721
29	1	0	-8.137856	1.160551	1.727992

**2**A000018_en_		Standard Orientation (A.U.)			
Center	Atomic	Atomic	X	Y	Z

number	number	Type			
0	6	0	-13.001051	-0.780999	-0.806964
1	6	0	-13.499608	1.728992	-0.37704
2	7	0	-15.987682	2.439209	-0.164767
3	6	0	-18.047295	0.83494	-0.314862
4	6	0	-17.310778	-1.796561	-0.790459
5	7	0	-14.952868	-2.484398	-1.0172
6	6	0	-11.588122	3.830069	0.000469
7	6	0	-10.390689	-1.886115	-1.042755
8	8	0	-20.233766	1.629297	-0.081613
9	6	0	-19.413708	-3.72126	-0.961447
10	6	0	-18.607791	-6.136313	-2.329057
11	6	0	-10.21427	3.584434	2.535986
12	6	0	-20.423467	-4.302366	1.711648
13	8	0	-9.887448	3.808235	-2.092626
14	1	0	-16.413286	4.28728	0.130726
15	1	0	-12.638456	5.626431	0.007194
16	1	0	-9.887735	-2.950193	0.665151
17	1	0	-10.348103	-3.220464	-2.623936
18	1	0	-8.972439	-0.429368	-1.36449
19	1	0	-20.95601	-2.818825	-2.01465
20	1	0	-17.095319	-7.099954	-1.29955
21	1	0	-20.216691	-7.429101	-2.486462
22	1	0	-17.909832	-5.726464	-4.233495
23	1	0	-8.906298	5.170512	2.789871
24	1	0	-11.57051	3.619549	4.097782
25	1	0	-9.143151	1.820394	2.628857
26	1	0	-21.047411	-2.573839	2.657282
27	1	0	-18.94845	-5.190492	2.863836
28	1	0	-22.030543	-5.603314	1.603884
29	1	0	-8.534791	4.985234	-1.70108

**2**A000019_en_		Standard Orientation (A.U.)			
Center number	Atomic number	Atomic Type	X	Y	Z
0	6	0	-14.37447	1.848785	-0.546694
1	6	0	-12.72839	0.142574	0.511677
2	7	0	-13.563419	-2.289704	0.828241
3	6	0	-15.938377	-3.18427	0.180329
4	6	0	-17.550408	-1.213474	-0.912103
5	7	0	-16.766824	1.107236	-1.225433
6	6	0	-10.010707	0.630634	1.3189
7	6	0	-13.745569	4.588276	-1.020414
8	8	0	-16.535094	-5.41701	0.530467
9	6	0	-20.215311	-1.958323	-1.620244
10	6	0	-21.836802	-2.205036	0.790548
11	6	0	-8.193561	0.43349	-0.924237
12	6	0	-21.418033	-0.134562	-3.512534
13	8	0	-9.739849	2.947754	2.657565
14	1	0	-12.392102	-3.591712	1.615211
15	1	0	-9.514089	-0.832887	2.699111
16	1	0	-11.957061	4.832925	-2.035796
17	1	0	-15.241548	5.410711	-2.180232
18	1	0	-13.629417	5.669107	0.741476
19	1	0	-20.094106	-3.846222	-2.471144

20	1	0	-23.739891	-2.864575	0.310635
21	1	0	-20.995215	-3.552611	2.112447
22	1	0	-22.007614	-0.370229	1.737331
23	1	0	-6.25128	0.715741	-0.273423
24	1	0	-8.331886	-1.427208	-1.816278
25	1	0	-8.630341	1.861592	-2.358475
26	1	0	-23.30692	-0.802121	-4.033326
27	1	0	-20.282334	0.021705	-5.235316
28	1	0	-21.596189	1.760344	-2.70358
29	1	0	-9.4711	4.286284	1.43499
